# Reduced stomatal density improves water-use efficiency in grapevine under climate scenarios of decreased water availability

**DOI:** 10.1007/s00299-025-03577-9

**Published:** 2025-08-07

**Authors:** Umar Shahbaz, Pierre Videau, Emma Coulonnier, Carla Papon, David Navarro-Payá, Alvaro Vidal Valenzuela, José Tomás Matus, Mickael Malnoy, Olivier Zekri, Fabio Fiorani, Michele Faralli, Lorenza Dalla Costa

**Affiliations:** 1 Research and Innovation Centre, Fondazione Edmund Mach, San Michele All’Adige, Italy; 2 Center Agriculture Food Environment (C3A), University of Trento, 38098 San Michele All’Adige, Italy; 3 Laboratoire Novatech Mercier, Le Champ Des Noëls, 85770 Le Gué de Velluire, France; 4 Institute for Integrative Systems Biology (I2SysBio), Universitat de València-CSIC, 46980 Paterna, Valencia Spain; 5 Institute of Bio- and Geo-Sciences, Forschungszentrum Jülich GmbH, 52425 Jülich, Germany

**Keywords:** *Vitis* spp*.*, Epidermal patterning factor like 9, Stomagen, Stomatal density, CRISPR/Cas9, Gas exchange, Water stress, Climate change

## Abstract

**Key message:**

The grapevine *VviEPFL9*-2 paralog is specifically expressed during leaf expansion and its knockout provide a phenotype with superior adaptation to environmental stresses via reduced stomatal density.

**Abstract:**

In *Arabidopsis* stomatal initiation relies on the transcription factor SPEECHLESS, which is positively regulated by AtEPFL9, a peptide of the epidermal patterning factor family. In grapevine, two *EPFL9* paralogs exist but despite a structural similarity, their specific function remains unclear. In this study, we investigated their distinct functional roles and the extent to which reduced stomatal density (SD) may be beneficial for grapevine in terms of water use. We combined expression analysis of the two paralogs in untreated and ABA-treated leaves with the functional characterization of the two genes using grapevine *epfl9*-1 and *epfl9*-2 mutants. A physiological analysis of *epfl9*-2 mutants under different environmental conditions was also performed. We showed that *VviEPFL9*-1 is exclusively expressed in leaf primordia, whereas *VviEPFL9*-2 plays a predominant role in fine-tuning SD during the leaf expansion. An *epfl9*-2 mutant line with 84% lower SD than wild type, exhibited a significant improvement in intrinsic water-use efficiency under both well-watered and water-stressed conditions, with little trade-off in photosynthesis. When the reduction in SD was close to 60%, photosynthetic rate and stomatal conductance were comparable to WT. Our results provide compelling evidence that *VviEPFL9*-2 knockout determines a significant reduction in stomatal density without a major impact on photosynthesis which may help optimize the adverse impacts of climate change on viticulture.

**Supplementary Information:**

The online version contains supplementary material available at 10.1007/s00299-025-03577-9.

## Introduction

One of the most important factors affecting plant growth and productivity in both ecological and agricultural environments is water availability (Allen et al. 2010). In recent years, water stress has been responsible for greater annual crop yield losses compared with those caused by pathogens. Moreover, the projections indicate that freshwater availability for agriculture will decrease in future while demand for arable land will increase (Gupta et al. 2020). Plants have evolved a multitude of strategies involving both shoot and root tissues to limit the detrimental effects of water stress (Rodrigues et al. 2019b). One of the most crucial mechanisms is the regulation of stomata, which are the microscopic pores on the leaf surface that control gaseous exchange between the plant and the atmosphere (Hetherington & Woodward 2003). Two types of stomatal responses to water deficit are known: a short-term response that operates on the time scale of minutes to hours by controlling the closure of the stomatal pore, and a long-term response on the time scale of weeks based on the modulation of stomatal density and size (Hamanishi et al. 2012; Franks & Casson 2014; Carvalho et al. 2016). Stomatal closure relies on the activation of a slow anion channel 1 (SLAC1) in guard cells (Li et al. 2022) induced by the accumulation of the phytohormone abscisic acid (ABA) (Deng et al. 2022). This phytohormone is also implicated in the long-term regulatory mechanisms of stomatal development. Indeed, evidence indicates that ABA suppresses the formation of stomata through the deactivation of the stomatal master regulator SPEECHLESS (SPCL) via the SnRK2 kinase acting with a specific phosphocode (Yang et al. 2022). Besides the central role of ABA in regulating stomatal development (Chater et al. 2014), other phytohormones, including brassinosteroids and auxin, play a role in this process (Gudesblat et al. 2012; Zhang et al. 2014; Nolan et al. 2023), with distinct hormonal circuits or in a crosstalk manner, in some cases tissue-specific (Wei et al. 2021). In addition to soil and air moisture, the formation of guard cells and their opening/closing movements are controlled by many other environmental cues. This indicates that stomata are highly regulated hubs for the fine-tuning of photosynthesis and transpiration to different climatic conditions. Recently, there were insights into the CO_2_-induced control of stomatal movements (Lopez et al. 2024) and into the molecular components of the signaling cascade linking light stimulus and heat stress to stomatal development (Samakovli et al. 2020; Wang et al. 2021).

With regard to stomatal morphogenesis, it has been demonstrated that in dicots it occurs via a series of asymmetric divisions in the leaf epidermis that are followed by a single symmetric division in a specialized epidermal stomatal lineage cell, which is committed to forming two guard cells (Bergmann et al. 2004). Pioneering studies in *Arabidopsis thaliana* have shown that small cysteine-rich peptides (CRPs), called epidermal patterning factors (EPFs), are responsible for regulating stomatal density and spacing in leaves (Doheny-Adams et al. 2012; Franks et al. 2015; Lee et al. 2015; Hepworth et al. 2015). EPFs are highly conserved across a wide range of higher plants (Lu et al. 2019; Karavolias et al. 2023) and three members of this family are essential for the formation of stomata: EPF1, EPF2, and Epidermal Patterning Factor Like 9 (EPFL9) (Marshall et al. 2011). EPFL9, also known as Stomagen, is a 45-aa peptide in its mature form, with three intramolecular disulfide bonds formed by the six cysteines present in the functional domain (Kondo et al. 2010). It is produced by leaf mesophyll cells and secreted into the epidermis via the apoplastic fluid, where it acts locally to promote stomatal differentiation (Zeng et al. 2020). This short-range signaling peptide works antagonistically to EPF1 and EPF2 by binding to the same heterodimeric receptor formed by ERECTA and TOO MANY MOUTHS (Marshall et al. 2011). While EPF1 and EPF2 activate the receptor complex, EPFL9 inactivates it, preventing the final destabilization of the essential transcription factors involved in the formation of stomata, namely SPCL and MUTE. Indeed, a mitogen-activated protein kinase (MAPK) cascade consisting of YODA, MKK4, MKK5, MPK3, and MPK6 transduces the signal from the activated receptor complex to the transcription factors SPCL and MUTE, inactivating them (Shpak 2013; Meng et al. 2013). Recent work has added a new piece to this complex puzzle of signaling cascades: it has been shown that MPK6 and MPK3 in mesophyll cells repress the expression of EPFL9 (Wu et al. 2024).

Genetic engineering and functional analyses have been instrumental in elucidating the function of EPF family peptides in a multitude of species. Insights were achieved through a combination of approaches, including gene overexpression, downregulation (RNA interference) and knock-out (CRISPR/Cas). The antagonistic role of EPF1 and EPF2, on one side, or that of EPFL9 on the other side, has been demonstrated primarily in cereals such as rice (Yin et al. 2017; Caine et al. 2019; Lu et al. 2019; Mohammed et al. 2019; Karavolias et al. 2023), barley (Hughes et al. 2017) and bread wheat (Dunn et al. 2019), but also in rapeseed (Jiao et al. 2023), sorghum (Ferguson et al. 2024), poplar (Jiao et al. 2022), and grapevine (Clemens et al. 2022). Evidence on the role of these peptides in stomata regulation has also been obtained for potato (Wang et al. 2020) and apple (Zhao et al. 2022). Interestingly, some members of the EPF family have been shown to have novel functions. Mohammed and colleagues (Mohammed et al. 2019), according to their observations in *OsEPF1* overexpressing rice plants, postulated an involvement of EPF1 in root cortical aerenchyma formation and recently, Guo and colleagues (2023) proposed that EPFL9, in combination with EPFL6 and EPFL7, contributes to rice panicle architecture (Guo et al. 2023).

In contrast to *Arabidopsis*, which has a single *AtEPFL9* gene in its genome (AT4G12970), EPFL9 was found to be duplicated in many crops. Paralogous genes have been found in cereals such as maize (Hepworth et al. 2018), rice (Lu et al. 2019), wheat (Jangra et al. 2021), sorghum (Karavolias et al. 2023), in annual and biennial dicots such as sunflower and carrot (Karavolias et al. 2023) and in fruit trees like apple (Zhao et al. 2022) and grapevine (Clemens et al. 2022). An in-depth investigation was recently conducted in rice by Karavolias and colleagues (2023), who elucidated the distinctive abilities of the two *OsEPFL9* paralogues in determining leaf stomatal density in productive rice plants. Indeed, the knockout of *OsEPFL9* and its paralog *OsEPFL10* resulted in stomatal density reductions of 75% and 20%, respectively, compared to the wild type. However, while *epfl9* rice mutants exhibited dramatic declines in stomatal conductance that were detrimental to carbon assimilation and thermoregulation, *epfl10* mutants were able to maintain high yield and efficient evaporative cooling. This work highlighted the importance of exploring in depth whether the EPFL9 paralogs may have a functional divergence in crops and how their manipulation may affect overall plant physiology. In grapevine two paralogs of *EPFL9* were found, *VviEPFL9-1* and *VviEPFL9-2*, sharing 82% of similarity in the protein sequence of their C-terminal functional domain (Clemens et al. 2022). In our previous research (Clemens et al. 2022), we focused specifically on *VviEPFL9-1* and showed that it plays a role in determining stomatal density, but only in small leaves of very young plants (1–2 months old). Accordingly, the difference in whole plant transpiration between *epfl9-1* mutants and wild-type was no longer significant in plants that were cultivated for several months in a greenhouse. The objective of the present study is to address the following research questions: (i) Do the two paralogs undergo differential transcriptional regulation during grapevine leaf development? Are there differences in greenhouse and field conditions? (ii) Do the transcript levels of the two paralogs respond to an ABA treatment or to water stress? (iii) What is the impact of the knock-out of *VviEPFL9*-2 on the stomatal density of fully expanded leaves? For a better comprehension of the distinct functional role of the two paralogs a comparison was made of stomatal density and morphology in *epfl9*-1 and *epfl9*-2 mutant plants of the same age and similar size. (iv) To what extent can a reduced stomatal density be effective in improving water use in well water conditions, and what are the consequences for photosynthesis? *epfl9*-2 mutants were also tested under light and CO_2_ curves and high VPD. (v) Finally, can a reduced stomatal density be beneficial for grapevine to better respond to conditions of water stress?

## Materials and methods

### Plant material for *VviEPFL9-1* and *VviEPFL9-2* transcripts profile and samples collection

For greenhouse plant material, biological replicates of in vitro healthy developed plants of ‘Sugraone’, ‘Syrah’ and ‘Cabernet Sauvignon’ were acclimatized in the greenhouse using 0.25 L plastic pots with three holes in the bottom to allow for water drainage, filled with a similar amount of growing substrate Extra quality—Semina 80 (TerComposti, Calvisano, Italy) and covered by parafilm on the top. The plants were kept in a growth chamber (PPFD 100 ± 20 µmol m^−2^ s^−1^, 24 °C, 16/8 light/dark photoperiod) and after one week, holes were gradually made in the parafilm over the course of two weeks. After 15 days, plants were repotted into 0.75 L pots containing growing substrate (Extra quality—Semina 80, TerComposti, Calvisano, Italy). Pots were kept in the same growth chamber for 10 days before moving to the greenhouse. In the greenhouse, plants were grown under natural light supplemented, when necessary, by a high-pressure sodium lamps system (PPFD 200–250 µmol m^−2^ s^−1^) with a 16-h/8-h light–dark photoperiod. For field plant material, ‘Sugraone’, ‘Syrah’ and ‘Cabernet Sauvignon’ cultivated in the field of the FEM grape germplasm collection (ITA362), which is situated in San Michele all'Adige, Italy (46°10ʹ53ʹʹN, 11°7ʹ2ʹʹE), were used. The three varieties were grafted onto ‘Kober 5BB’ rootstock and planted in 2003 in a sandy-loamy soil (five vines per variety). Vines of all varieties had a between-row and within-row spacing of 2 m × 1 m and row orientation was north–south. The vines were managed using the Guyot pruning method and trained using the VSP trellis system. Vines were irrigated during the growing seasons via drip irrigation according to evapotranspiration requirements and via farm irrigation scheduling. Environmental data were obtained from the San Michele all’Adige weather station (205 m a.s.l., 46.190023N, 11.134712E). As for samples collection, leaf primordia and leaves at increasing distance from the apex of 4 biological replicates of the cultivars ‘Sugraone’, ‘Syrah’ and ‘Cabernet Sauvignon’ were collected from plants derived from in vitro culture and grown in the FEM greenhouse for 3 months or from the field (May and June 2022). The leaf area is shown in Fig. S1.

### Experimental design of ABA treatment and sample collection

Sixteen biological replicates of ‘Cabernet Sauvignon’ and ‘Syrah’ plants deriving from in vitro culture and cultivated in the FEM greenhouse for 3 months were utilized. A solution containing 100 mg/L of ABA (370 µM) and 0.05% Tween 20 was prepared in a volume of 0.6 L and transferred into a 1.5 L plastic pressure sprayer. Twelve plants of each cultivar were thoroughly sprayed from the apex to the base to ensure uniform coverage. Four plants were used as an untreated control. Leaf primordia and the first expanded leaf (a leaf with a main vein around 6 cm long and an area of around 15–20 cm^2^) were collected from 4 biological replicates for each following groups: control, 2 h post treatment (hpt), 24 hpt and 48 hpt. After collection, the detached leaf and leaf primordium were immediately frozen in liquid nitrogen for subsequent RNA extraction.

### Gene expression analysis

Total RNA was extracted from the material collected from the plants and immediately frozen in liquid nitrogen. The Spectrum Plant Total RNA Kit (Sigma–Aldrich, St. Louis, MO, USA) was used to extract RNA. Extracted RNA was measured using the NanoDrop ND-8000 spectrophotometer (NanoDrop Technologies, Wilmington, DE, USA). After DNase treatment, 1 μg of RNA was reverse-transcribed into cDNA utilizing SuperScript III Reverse Transcriptase (Invitrogen, Carlsbad, CA, USA) with oligodT primers, adhering to the manufacturer's guidelines. The real-time PCR was conducted using the CFX96 equipment (Bio-Rad, Hercules, CA, USA) in a 12.5 μL volume including 1 × Sybr green TB Green Premix Ex Taq II (Takara, Kusatsu, Japan), 0.5 μM primers (Table S1), and 1 μL of diluted cDNA (1:10). An initial denaturation stage at 95 °C for 5 min was succeeded by 40 cycles at 95 °C for 10 s and 60 °C for 30 s. To identify non-specific amplification in cDNA samples, a melting curve analysis was conducted as follows: 95 °C for 10 s, 65 °C for 5 s, followed by a gradual temperature increase (0.5 °C/s) to 95 °C with continuous monitoring. Glyceraldehyde 3-phosphate dehydrogenase (GAPDH) (Reid et al. 2006) and actin (ACT) (Gatto et al. 2008) were used as housekeeping genes for determining a normalization factor. For each biological replicate of each plant line, two technical replicates were conducted on a single plate, and the data were analyzed using Bio-Rad CFX Manager 3.0 software.

### RNAseq database analysis

An extensive library of publicly available RNA-seq datasets was obtained from SRA (NCBI) and the automatic classification system described in Santiago et al. (2024) was used to standardize and classify tissue metadata terms. A total of 8454 runs (3663 leaf, 182 flower, 3613 berry, 45 seed, 12 embryo, 95 ovary, 330 root, 206 bud, 192 dormant bud and 116 floral bud runs) was obtained and used to examine gene expression (Table S2). Additional manual curation of metadata was used to identify a subset of water stress related studies (997 runs) where stress tolerant and susceptible cultivars were additionally marked (Table S3).

### Stomatal density (SD) measurement

Stomatal density was measured on the same leaves used for gene expression analysis (after the imprints leaves were immediately frozen in liquid nitrogen for gene expression analysis). A small layer of dental paste Affinis Precious (Coltene, Brianza, Italy) was applied to the abaxial surface of the leaf using an Affinis dispenser (Coltene, Brianza, Italy). Upon drying, the dental paste layer was carefully removed from the leaf, horizontally positioned and treated with a transparent gel nail polish to obtain a leaf imprint. The imprint was then placed onto a microscope slide and imaged using an inverted optical microscope (Motic AE31 Nanovision) at both 10 × and 20 × magnifications. For each leaf, three imprints (two imprints for the analysis shown in Fig. 7) were taken, and for each imprint, three random images were taken. Image analysis and stomatal counting were conducted manually using the ImageJ software (https://imagej.nih.gov/ij/).

### Stomatal size (SS) measurement

Stomatal pore length was measured using ImageJ software on the same images used for stomatal density analysis. A total of 36 images per genotype were analyzed for pore length measurement. From each image, eight randomly selected stomata were manually measured, resulting in a total of 288 stomata per genotype for pore length analysis.

### Vector design, gene transfer and regeneration of edited and overexpressing lines

Edited plants knocked-out in the *VviEPFL9-1* gene were generated in the study carried out by Clemens et al. (2022) while edited plants knocked-out in the *VviEPFL9-2* gene were produced in the current study by using two CRISPR/Cas9 binary vectors purchased from DNA Cloning Service (Hamburg, Germany). In addition to the common CRISPR/Cas9 elements and *nptII* gene, each of these vectors carried a specific guide RNA designed by us with CRISPR-P 2.0 software (http://crispr.hzau.edu.cn/cgi-bin/CRISPR2/CRISPR) and validated for RNA secondary structure (http://rna.tbi.univie.ac.at/cgi-bin/RNAWebSuite/RNAfold.cgi): (1) sgRNA_937EPFL9-2 CATTCATTGTAAGTGCAGGT, recognizing a 20 bp region in the third exon of *VviEPFL9-2* (reverse strand); (2) sgRNA_938EPFL9-2 GACTGATGATTGGATCCACC, recognizing a 20-bp region in the third exon of *VviEPFL9-2* (forward strand, upstream and adjacent to sg937). *VviEPFL9-*2 overexpressing lines were generated using a pK7WG2 binary vector (Karimi et al. 2002) with the *VviEPFL9*-2 gene under the control of the CaMV-35S promoter. The *VviEPFL9*-2 gene (Vitvi05_01chr07g18680; 656 bp total length excluding UTR regions, see Fig. 3a for the gene structure) was amplified from genomic DNA of ‘Sugraone’ using the primers indicated in Table S1, cloned initially into pGEM-T easy vector (Promega, Milano, Italy) and finally into gateway vectors (sequentially pDONR 202 and pK7WG2 by BP and LR clonase reactions) according to the manufacturer's instructions. Gene transfer was carried out by means of *Agrobacterium tumefaciens* (*A.t*.) on embryogenic calli of the varieties ‘Sugraone’, ‘Syrah’ and of the rootstock ‘Kober 5BB’ according to the procedure described by (Dalla Costa et al. 2022). Several regenerated plants were analyzed by PCR for the initial screening of *SpCas9* or *nptII* integration in the edited or overexpressing lines, respectively. PCR was carried out in 20 µl final volume containing 1 × PCR BIO (Resnova, Rome, Italy), 0.5 µM of each primer (Table S1) and 20 ng of genomic DNA. DNA was extracted from freshly frozen leaf tissue (approximately 100 mg) using Nucleospin Plant II kit (Macherey–Nagel, Düren, Germany) following the manufacturer’s instruction, quantified using Nanodrop 8800 (Termo Fischer Scientific, Waltham, MA, USA) and diluted to a final concentration of 20 ng/µL. In vitro lines and the WT control were propagated in sterile baby jars in WP medium (McCown and Lloyd 1981) and grown in a climatic chamber at 100 photosynthetic photon flux density (PPFD) ± 20 (µmol m^−2^ s^−1^), 24 °C and a 16/8 light/dark photoperiod.

### On-Target and off-target editing evaluation

In the grapevine lines which integrated T-DNA, a region of the gene *VviEPFL9*-2 containing the site targeted by the sgRNA/Cas9 complex was amplified with primers *VviEPFL9*-2_fw and *VviEPFL9*-2_rv (Table S1) both elongated with overhang Illumina adapters. PCR was carried out in 20 μL final volume containing 1 × PCR BIO (Resnova, Rome, Italy), 0.4 μM of each primer (Table S1) and 30 ng of genomic DNA. The Illumina library was sequenced on an Illumina MiSeq (PE300) platform at the Sequencing Platform Facility of Fondazione Edmund Mach (San Michele all’Adige, Italy). CRISPResso2 pipeline4 (Clement et al. 2019) was used to process the raw paired end reads with default parameters and to visualize the mutation profiles in the target sequences. The analysis of off-target sites was performed in-silico using the PN40024 reference genome. For the off-target site differing from the sgRNA by only two mismatches, a PCR was performed in 25 μL final volume containing 1 × PCR BIO (Resnova, Rome, Italy), 0.5 μM of each primer (Table S1) and 30 ng of genomic DNA. Amplification products were checked on an agarose gel, purified using CleanNGS magnetic beads (CleanNA, Waddinxveen, Netherlands) and sequenced by Sanger sequencing (FEM Sequencing Platform Facility). The sequencing results were analyzed using the Blast online tool.

### Transgene copy number (CN) quantification

Real-time PCR quantification of *SpCas9* CNs in grapevine lines was performed in a 96-well plate on a C1000 thermal cycler (Bio-Rad, Hercules, USA) equipped with a CFX96 real-time PCR detection system (Bio-Rad,Hercules, USA). The real-time PCR reaction was performed in a final volume of 12.5 μL containing 1 × Sybr green TB Green Premix Ex Taq II (Takara, Kusatsu, Japan), 40 ng of genomic DNA and 0.4 μM primers (Table S1). The thermal protocol was as follows: polymerase activation for 30 s at 95 °C, 40 cycles of denaturation for 5 s at 95 °C and annealing/extension for 15 s at 58 °C, followed by the melting curve protocol consisting of 95 °C for 10 s, 65 °C for 5 s and a stepwise T increase (0.5 °C/s) up to 95 °C with a continuous detection. The primers used to amplify the endogenous grapevine gene *VviCHI* and the exogenous gene *SpCas9* were indicated in Table S1. The standard curves, one for the endogenous *VviCHI* and one for the *SpCas9* transgene were generated with a pGEM-T easy plasmid (Promega, Madison, Wisconsin, USA) containing specific fragments of the genes to be amplified, and consisted of four points, starting from 10^6^ plasmid molecules with a serial dilution of 1:5. For each sample, the transgene CN was calculated using the following formula: (total transgene copies/total endogenous gene copies) × 2. For each sample, the total copies of the transgene and of the endogenous gene were obtained from the standard curves based on the mean values of the quantification cycles (Cq).

### T-DNA integration site identification

T-DNA integration points (IP) were determined following the method described in Dalla Costa et al*.* (2020) and primers reported in Table S1. The library was sequenced by Illumina MiSeq (PE300) platform at the Sequencing Platform Facility of Fondazione Edmund Mach (San Michele all’Adige, Italy). The putative genomic regions identified were validated by PCR amplification. PCR was performed in 20 µL final volume containing 1 × PCR BIO (Resnova, Rome, Italy), 40 ng of genomic DNA and 0.5 µM of the primers reported in Table S4. Amplification products were checked on agarose gel, purified using PureLink Quick Gel Extraction (Invitrogen, Carlsbad, CA, USA) and sequenced by Sanger sequencing (FEM Sequencing Platform Facility). Sequencing outputs were analyzed with the Blast sequence server (using the PN40024 40X genome assembly, associated to v4.3 annotation) available online at the Grapedia website (https://grapedia.org/genomes/).

### Plant material used for physiological analysis

The gas exchange analyses were carried out on five biological replicates of ‘Sugraone’ WT and *epfl9*-2 selected mutant lines, namely Su_*epfl9*-2a and Su_*epfl9*-2b, 70 ± 20 cm tall, deriving from in vitro culture and cultivated in a greenhouse at PPFD 100 ± 20 µmol m^−2^ s^−1^, 25 °C, 50% RH, in pots (23.5 × 16 × 16 cm) containing 1250 g of the substrate Extra quality—Semina 80 (TerComposti, Calvisano, Italy).

### Gas-exchange measurements

Gas-exchange measurements including CO_2_ assimilation rate at saturating PPFD (*A*_sat_), stomatal conductance (*g*_s_) and intrinsic water-use efficiency (_i_*WUE*) calculated as *A*_sat_/*g*_s_, as well as *A*/*Ci* curve, light response curve and vapor pressure deficit (VPD) curve were carried out using a portable infrared gas analyzer LI-6800 (LI-COR Biosciences Inc. Lincoln, NE, USA) and a 2-cm^2^ leaf cuvette with an integrated blue-red LED light source. In the cuvette the flow rate was set at 750 μmol s^−1^, the temperature of the leaf at 25 °C, the light intensity at 1000 PPFD (μmol m^−2^ s^−1^), the CO_2_ concentration at 420 ppm, the VPD at 1.5 kPa, and the humidity at 60%. To ensure that the gas exchange reaches a steady state, a 5-min steady state was set at every environmental parameter step change prior to taking the measure. The measurements were conducted from March to May 2024 at the research greenhouse of IBG2: Plant Sciences, Forschungszentrum Jülich (FZJ) in Germany (50°55′17.36″N, 6°21′45.61″E) on fully expanded leaves of five biological replicates of ‘Sugraone’ WT, *epfl9*-2a and *epfl9*-2b mutant plants.

### A/Ci curve

The response of photosynthetic assimilation rate (*A*) to sub-stomatal CO_2_ concentrations (*Ci*) was measured between 9:00 AM and 12:00 AM. The CO_2_ baseline concentration in the leaf cuvette was set at 400 ppm and then reduced and raised in a sequential manner as follows: 300, 200, 100, 50, 0, 400, 600, 800, 1000 and 1200 ppm. During the experiment the PPFD was set at a constant value of 1000 μmol m⁻^2^ s⁻^1^. The values of the maximum photosynthetic carboxylation rate of Rubisco (*V*_cmax_) and the maximum rate of electron transport demand for Ribulose 1,5-bisphosphate (RuBP) regeneration (*J*_max_) were computed as proposed by Duursma (2015). A non-rectangular hyperbola model was used to fit the A/Ci curve (Sharkey et al. 2007).

### Light response curve

The response of photosynthetic assimilation rate (A) to light variations was measured between 9:00 AM and 3:00 PM. The light intensity (PPFD) was first increased to 1500 μmol m^−2^ s^−1^ and then gradually decreased to the following levels: 1300, 1100, 1000, 900, 800, 600, 400, 300, 100, 50 and 0 μmol m^−2^ s^−1^. A modified rectangular hyperbola model was used to fit the light response curve (Ye 2007).

### VPD curve

The transpiration rate (E) was measured under the following VPD_air values in the leaf cuvette: 0.5; 0.8; 1.0; 1.3; 1.5; 1.7; 2.0; 2.5; 3.0; 3.5; 3.7 and 4.0 kPa. The VPD_air values were calculated using chamber saturation vapor pressure (SVPcham) and relative humidity (RHcham) parameters. A constrained two-segment linear regression model was used to determine the VPD threshold at which the relationship between transpiration and VPD changes, reflecting stomatal regulation (Oren et al. 1999).

### Plant material and experimental design of water stress experiment 1

A water stress experiment was conducted using ten biological replicates of ‘Sugraone’ WT, Su_*epfl9*-2a and Su_*epfl9*-2b mutant plants, 80 ± 10 cm tall, deriving from in vitro culture and cultivated in a greenhouse under photoperiod 14/10 h, average PPFD 100 ± 20 µmol m^−2^ s^−1^, 25 °C, 45% RH, in pots (23.5 × 16 × 16 cm) containing 1250 g of the substrate Extra quality—Semina 80 (TerComposti, Calvisano, Italy). At the beginning of the experiment plants were covered on the top of the pot with aluminum foil. The experiment followed a randomized complete block design (RCBD) with two water treatments (well-watered and water stress). Each block contained six pots, with plants randomly assigned to well-watered or water stressed conditions. Well-watered plants were manually irrigated with 500 mL of water per pot every second day to maintain soil moisture content. Water stress was imposed on half of the biological replicates of the WT and mutant lines (*n* = 5), by withholding the water for a period of 14 days. *A*_sat_, *g*_s_ and _i_*WUE* were measured at 1, 4, 8, 11, 14 days after stress application (DASA) using a LI-6800 portable infrared gas analyzer (LI-COR Biosciences Inc. Lincoln, NE, USA) with the same parameters indicated in the “Gas-exchange measurements” section. The plant stress level was evaluated through the measurement of stem water potential on the fully expanded leaf at 10 days after stress application (DASA).

### Stem water potential

Stem water potential (Ψstem) was measured at midday (about 12 AM to 1 PM) by using a pressure chamber (Scholander Chamber, Model M-1000, PMS Instrument Company, Albany, OR, USA) on the fully expanded leaf after 10 days of water stress. The leaves were enclosed in plastic bags coated with aluminum foil for at least one hour before measurement to provide equilibration between the stem and leaf water potential.

### Plant material and experimental design of water stress experiment 2

A second water stress experiment was conducted using unequal number of biological replicates of WT, *epfl9*-2a and *epfl9*-2b mutant plants of three cultivars, ‘Sugraone’, ‘Kober 5BB’ and ‘Syrah’, and of ‘Sugraone’ *VviEPFL9*-2 overexpressing lines (Table S5) all derived from in vitro culture and acclimatized in greenhouse as described below. The plant height is reported in Fig. **S5**, the substrate used was Promix formula—coco grapevine (Premier Tech, Rivière-du-Loup, Canada) and the pot volume was 1 L (filled with 560 g substrate per pot). Three irrigation conditions were established: a control condition (well-water), a moderated and a severe stress condition. Due to the absence of a soil water potential measurement system and the variability of water requirements depending on uncontrolled temperature conditions, the irrigation days were not fixed but adjusted according to plant needs and visual soil observations (Fig. S6). Plants in the well-watered condition were irrigated regularly to maintain consistently moist soil. Under moderate stress condition, watering was only applied when the plants began to wilt, and the soil was dry. In the severe stress, irrigation was still applied but only when the soil was extremely dry. Irrigation consisted of 250 mL of tap water applied uniformly to all plants of the same genotype within the same condition. Plant height was measured from the base of the stem to the apex using a graduated ruler. An initial measurement was taken before the start of the experiment to serve as a baseline for comparison. A second measurement was taken after two weeks of well-watered irrigation for all plants (T0). After the start of the experiment, plant height was measured weekly (T1, T2, T3, T4).

### Statistics

Statistical analyses were performed using R software (R Core Team 2020). ANOVA and post hoc comparisons were carried out to assess group differences. *P* values lower than 0.05 were considered significant.

## Results

### *VviEPFL9* paralogue genes are differentially regulated in grapevine

**Fig. 1:**
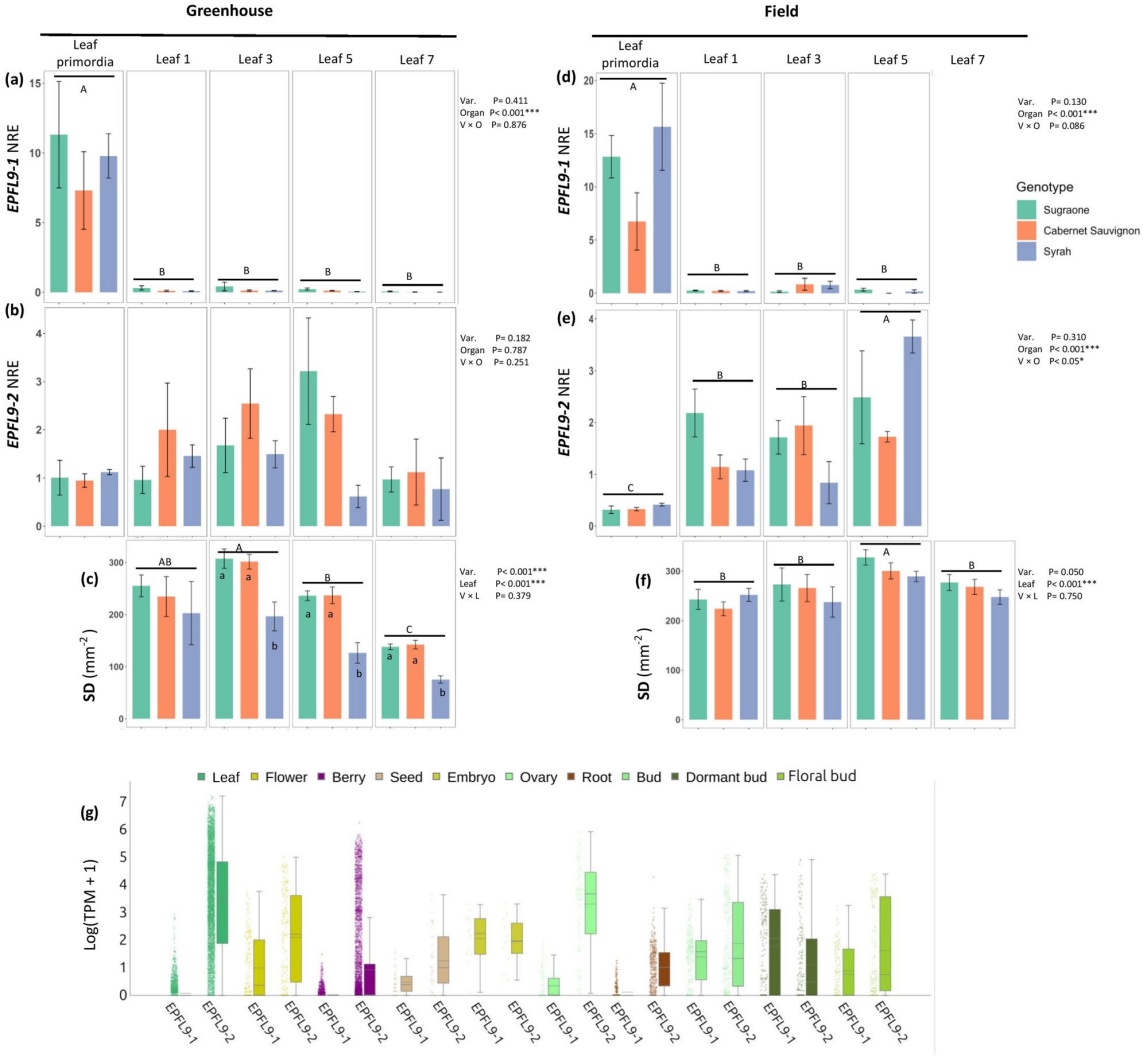
*VviEPFL9* paralogs expression and stomatal density (SD) profile. **a** *VviEPFL9*-1 and **b** *VviEPFL9*-2 transcript profile in leaf primordia and leaves at increasing distance from the apex (i.e., different leaf age) of greenhouse grown plants. **c** On the same leaves used for **a** and **b** stomatal density (SD) was calculated as the number of stomata per mm2. **d** *VviEPFL9*-1 and **e** *VviEPFL9*-2 transcript profile in leaf primordia and leaves at an increasing distance from the apex of field grown plants. **f** On the same leaves used for **d** and **e** stomatal density (SD) was calculated as the number of stomata per mm2. Bars show the mean ± SE (*n* = 3–4). Statistical significance was determined by two-way ANOVA followed by Tukey's post-hoc test (*P* < 0.05). (g) *VviEPFL9*-1 and *VviEPFL9*-2 expression across 8454 public Illumina-based transcriptomic runs classified and curated into different tissues (see Table S2 for the associated metadata of these runs)

*VviEPFL9* paralogs were initially identified in the v4.3 annotation associated to the PN40024 40X genome assembly and named *VviEPFL9*-1 (Vitvi05g01370) and *VviEPFL9*-2 (Vitvi07g04390). *VviEPFL9*-2 was not present in any other previous annotation, however, they are both retained in the T2T reference genome (v5.1 annotation) as Vitvi05_01chr05g20810 and Vitvi05_01chr07g18680, respectively. The gene expression patterns of *VviEPFL9*-1 and *VviEPFL9*-2 were examined in a panel of *Vitis vinifera* cultivars grown in the greenhouse: two wine cultivars, one considered to be a water conservative genotype, cv. ‘Cabernet Sauvignon’, and the other a typical anisohydric genotype, cv. ‘Syrah’, while the third was a table grape, cv. ‘Sugraone’. In all of them, *VviEPFL9*-1 was observed to be expressed in the leaf primordia and shoot apical meristem of the shoot tip, while its transcripts were undetectable in the assessed leaves (Fig. 1a). In contrast, *VviEPFL9*-2 was expressed in both the shoot tip and leaves (Fig. 1b) in all three genotypes, with a similar trend, namely a maximum expression in leaf 3 or 5, followed by a general decline in fully developed leaf 7. However, due to high variability among replicates, no significant differences were observed between genotypes and organs. Furthermore, an analysis of stomatal density was conducted on the same leaves used for gene expression analysis. While the stomatal density values of 'Sugraone' and 'Cabernet Sauvignon' were comparable at leaf stage 3 (307 vs 301 stomata/mm^2^), leaf stage 5 (236 vs 236 stomata/mm^2^), and leaf stage 7 (138 vs 142 stomata/mm^2^), those of ‘Syrah’ were statistically lower compared to the other two cultivars (*P* < 0.001; Fig. 1c).

The same characterization was performed on the same cultivars grown in experimental vineyards at the Edmund Mach Foundation. In general, the expression data for both genes exhibited similarities to those observed under greenhouse growth conditions. In particular, the expression of *VviEPFL9-*1 was only detectable in the shoot tip (Fig. 1d), while that of *VviEPFL9*-2 was observed in all leaves analyzed along the plant axis, although it was not possible to analyze leaf 7 due to the low amount of RNA and the high content of inhibiting compounds that make leaf 7 RNA unviable for further processing. It is worth noting that the genotype that shows more variation between the greenhouse and field is ‘Syrah’ (Fig. 1e). Furthermore, the analysis of stomatal density in field conditions showed that ‘Syrah’, ‘Cabernet Sauvignon’, and ‘Sugraone’ exhibited similar values throughout leaf development with the maximum stomatal density observed at leaf stage 5 (289, 300 and 328 stomata/mm^2^, respectively). (Fig. 1f).

Moreover, according to the reanalysis of publicly available transcriptomic datasets (Fig. 1g), *VviEPFL9*-2 exhibited a higher level of expression than *VviEPFL9*-1 in grapevine leaf tissue, while the differences in expression were relatively minimal in buds containing leaf primordia, and in the subclassification of dormant and floral buds. With regard to other tissues, *VviEPFL9*-2 exhibited higher expression levels than *VviEPFL9*-1 in berries, seeds, flowers, ovaries, and roots. Nevertheless, comparable expression levels were observed in embryo tissues (Fig. 1g).

**Fig. 2:**
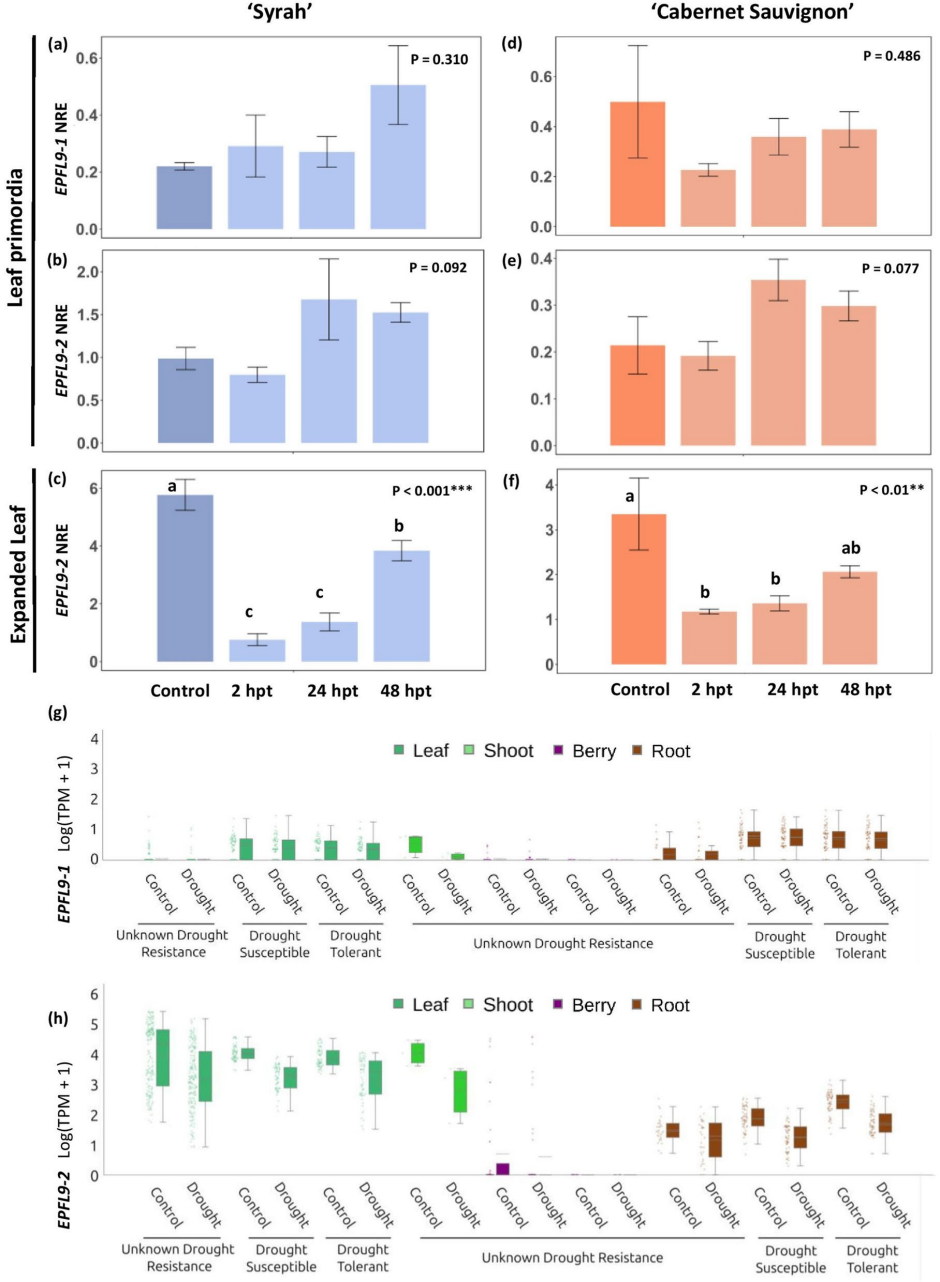
Effect of Abscisic acid (ABA) and water stress on the expression of VviEPFL9 paralogs in different organs. **a, d** Transcript profile of *VviEPFL9*-1 in leaf primordia and of *VviEPFL9*-2 in leaf primordia **b, e** and in the first fully expanded leaf **c, f** of respectively ‘Syrah’ and ‘Cabernet Sauvignon’ greenhouse grown plants treated with ABA. Samples were collected from untreated plants (control) and from treated plants at increasing time points after ABA treatment (hours post treatment, hpt). Bars show the means ± SE (*n* = 3–4). Statistical significance was determined by two-way ANOVA followed by Tukey's post-hoc test (*P* < 0.05). Expression of *VviEPFL9*-1 (**g**) and *VviEPFL9*-2 (**h**) across a set of 997 transcriptomic runs (both Illumina and ABI-Solid) related to water stress experiments in different organs (see Table S3 for the associated metadata of these runs)

We next examined how ABA and water stress regulates *VviEPFL9*-1 and *VviEPFL9*-2 expression. In both 'Syrah' and 'Cabernet Sauvignon' varieties, the expression of *VviEPFL9-1* and *VviEPFL9-2* in the shoot tip tissues was not modulated by the exogenous application of ABA (Fig. 2a,b and 2d,e). On the contrary, ABA treatment resulted in a significant repression of *VviEPFL9*-2 in the first fully expanded leaf (Fig. 2c,f; *P* < 0.001 for ‘Syrah’ and *P* < 0.01 for ‘Cabernet Sauvignon’). In both varieties, the expression levels exhibited a marked down-regulation at 2 h-post-treatment (hpt) with a reduction of approximately six-fold for 'Syrah' and 2.8-fold for ‘Cabernet Sauvignon’ compared to the control, achieving the lowest levels observed in the experiment. By 48 hpt, the expression began to recover, approaching pre-treatment values. We screened for the presence of cis-acting elements involved in abscisic acid responsiveness in the 2.5k bp promoter region of *VviEPFL9*-1 and *VviEPFL9*-2 using the PlantCARE tool for in silico analysis of promoters (Lescot et al. 2002). An ABRE motif (ACGTG sequence) is present at positions -1338 and -2287 (starting negative counting from the transcription start) in the *VviEPFL9*-1 promoter, while at position -326 in the *VviEPFL9*-2 promoter.

Moreover, the reanalysis of publicly available transcriptomic datasets of water stress experiments indicated that, in leaf, shoot and root tissues, *VviEPFL9*-2 expression appears to be repressed in conditions of drought compared to plants maintained under well-watered conditions (Fig. 2h). Consistent with the experimental data shown in Fig. 2a,d this modulation is not discernible for *VviEPFL9*-1 expression in leaves and roots (Fig. 2g).

### *VviEPFL9*-2 plays a major role in determining stomatal density in grapevine leaves

**Fig. 3:**
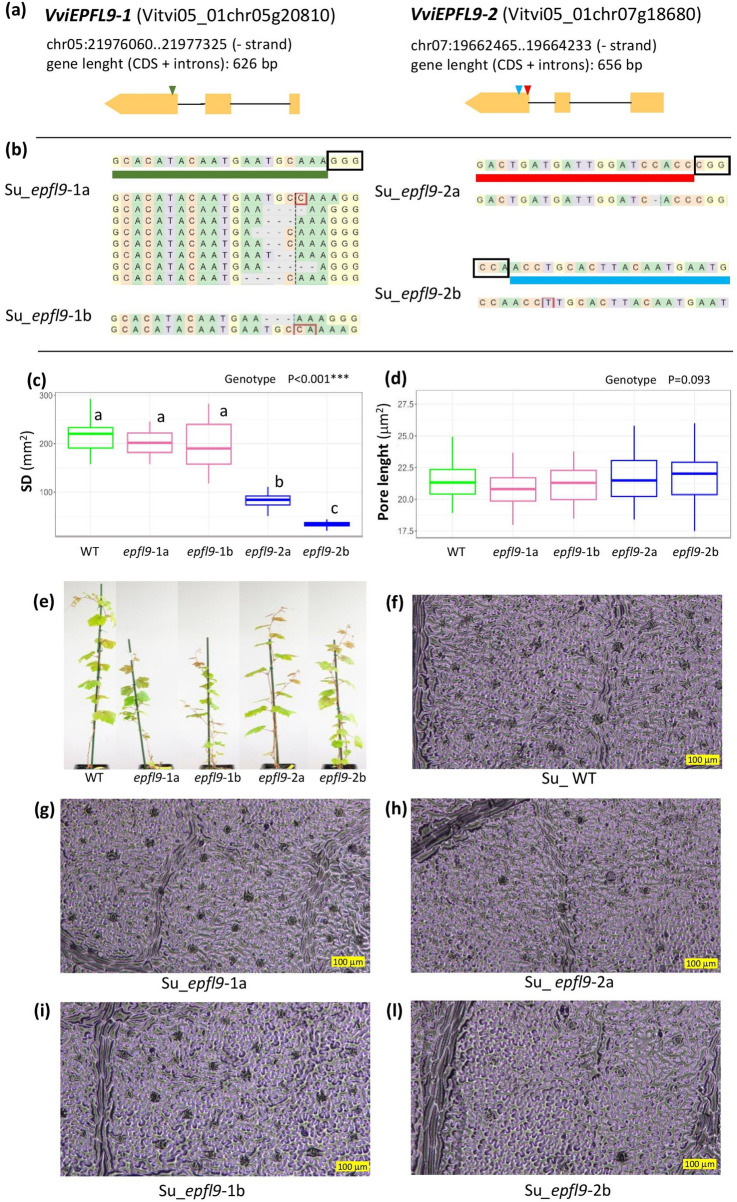
Characterization of stomatal anatomical features in Su_*epfl9*-1 and Su_*epfl9*-2 ‘Sugraone’ mutants. **a** Locus ID, gene position and gene structure of *VviEPFL9*-1 and *VviEPFL9*-2 according to the *Vitis vinifera* reference genome PN40024 T2T (v5.1 annotation). The sgRNA used to knock-out *VviEPFL9*-1 (Clemens et al. 2022) is indicated by a green triangle while the two sgRNAs used to knock-out *VviEPFL9*-2 (sg937EPFL9-2 and sg938EPFL9-2) are indicated by a blue and a red triangle, respectively. **b** Allele plot of the target site according to the analysis of the Illumina sequencing by CRISPResso2. The sgRNA was indicated with a colored line and the PAM site is indicated by a black box. **c** Stomatal density and **d** stomatal pore size evaluated for Su_WT, Su_*epfl9*-1 and Su_*epfl9*-2 mutants. For each mutant line and WT, 4 biological replicates were evaluated and, for each biological replicate, one fully expanded leaf (the 5th from the apex) was analyzed. Statistical significance was determined by one-way ANOVA followed by Tukey's post-hoc test (*P* < 0.05). **e** Selected biological replicates for each line and WT under analysis. **f-g-h-i-l** Images of nail polish imprints respectively from Su_WT, Su_*epfl9*-1a, Su_*epfl9*-2a, Su_*epfl9*-1b, Su_*epfl9*-2b

As illustrated in Fig. 3b, the ‘Sugraone’ mutants under analysis exhibited loss-of-function mutations in the third exon of the specific paralog. The target site of the transgenic line Su_*epfl9*-1a displayed a single base insertion and multiple patterns of deletion, whereas the Su_*epfl9*-1b line displayed an insertion of two bases and a deletion of three bases. This latter mutation does not cause a frameshift in the coding region as the other modifications do but leads to a deletion of a cysteine residue that is required for the formation of one of the three disulfide bonds necessary for the correct folding of the functional peptide (Clemens et al. 2022). In contrast, the *VviEPFL9-2* edited lines, Su_*epfl9*-2a and Su_*epfl9*-2b, which were obtained with sgRNA937 and gsRNA938, respectively, (indicated in blue and red in Fig. 3a), exhibited a homozygous mutation consisting of a single base deletion or a single base insertion, respectively.

The identification of the T-DNA genomic integration point for one Su_*epfl9*-1 and the two Su_*epfl9*-2 evaluated lines (Table S4) allows us to ascertain that the four lines originated from independent genetic transformation events (Fig. S2), thereby excluding the possibility that the observed plant phenotype may be derived from a modification other than the desired one in the target site (i.e., a “T-DNA positional effect”). In addition, it was decided to check for possible off-target regions containing less than three mismatches with respect to the target sequence. In fact, based on our experience, three mismatches are sufficient to exclude recognition and cutting by the sgRNA-Cas9 complex. According to the PN40024 v5 reference genome (PN40024 12X.2 assembly), one possible off-target was found for sgRNA937 on the *VviEPFL9*-1 gene, with only two mismatches with the target region. In the Su_*epfl9*-2b line, edited with sgRNA937, the off-target region did not show any mutation compared to the expected reference sequence. In the case of sgRNA938, the possible off-targets had at least 3 mismatches with the on-target and were not evaluated.

As shown in Fig. 3c,f,h,l, the stomatal density (SD) of mature leaves of Su_*epfl9*-2a and Su_*epfl9*-2b mutants was significantly reduced compared to WT, exhibiting a decrease in SD of 61 and 84%, respectively. In contrast, the SD of Su_*epfl9*-1a and Su_*epfl9*-1b was not different from that of WT (Fig. 3c,f,g,i). With respect to stomatal pore size, no significant differences were observed between WT and the *epfl9* mutants (Fig. 3d).

To further support our findings, we also edited *VviEPFL9*-2 in the cultivar 'Syrah' and in the rootstock ‘Kober 5BB’ (Table S4, Fig. S3). The mutants exhibited a significant reduction in SD compared to WT (Fig. 7a).

### A reduction in stomatal density induced by *VviEPFL9*-2 knock-out may not have a strong impact on photosynthesis, regardless of environmental conditions

**Fig. 4:**
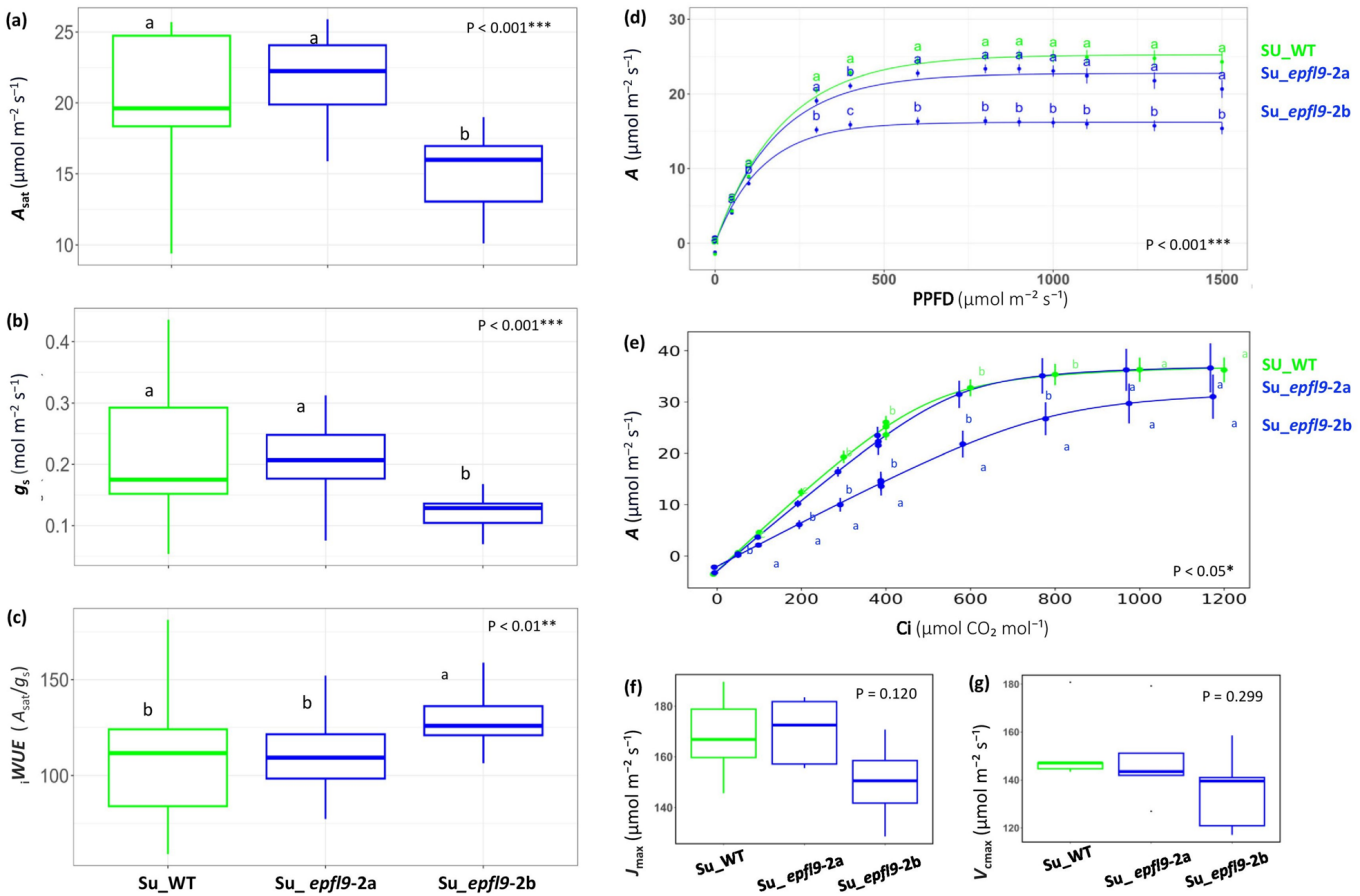
Photosynthetic traits in *epfl9*-2 mutants and WT. **a** CO_2_ assimilation rate at saturating light (*A*_*sat*_). **b** Stomatal conductance (*g*_s_), and **c** intrinsic water-use efficiency (*iWUE*) calculated as *A*_*sat*_/*g*_*s*_. **d** CO_2_ assimilation response to increasing light intensity (from 0 to 1500 µmol m⁻^2^ s⁻^1^ PPFD). **e** CO_2_ assimilation response (A) to increasing CO_2_ (Ci) (from 0 to 1200 µmol CO_2_ mol⁻.^1^). **f** Maximum electron transport rate for ribulose bisphosphate regeneration (*J*_max_) and **g** maximum velocity of Rubisco carboxylation (*V*_*cmax*_) calculated using *A/Ci* curves. Statistical significance was determined by one-way ANOVA followed by Tukey's post-hoc test (*P* < 0.05, *n* = 5)

To investigate how a reduction in stomatal density impacts on CO_2_ assimilation rate (*A*), stomatal conductance (*g*_s_), and intrinsic water use efficiency (_i_*WUE*), a physiological characterization was conducted on ‘Sugraone’ *epfl9-2* mutants. No significant differences were observed in *A* between Su_WT and Su_*epfl9*-2a while Su_*epfl9*-2b line showed a significantly decreased *A* value compared to WT (*P* < 0.001) (Fig. 4a) by up to 4 μmol m^−2^ s^−1^. Accordingly, the analysis of *g*_s_ and of _i_*WUE* (Fig. 4b and c) revealed significant differences between Su_*epfl9*-2b on one side and Su_*epfl9*-2a and Su_WT on the other (*P* < 0.001 and *P* < 0.01, respectively). Moreover, it was observed that, under conditions of varying light intensity, *A*_*max*_ was notably lower in the Su_*epfl9*-2b line compared to the Su_WT line (*P* < 0.01) (Fig. 4d). In contrast, the Su_*epfl9*-2a line exhibited a behavior similar to that of the WT line (Fig. 4d). Dark respiration (Rd), calculated from the light-response curve, exhibited significant differences (P < 0.01) between Su_WT (1.98 ± 0.21) and Su_*epfl9*-2b (1.47 ± 0.26), while an intermediate value was obtained for Su_*epfl9*-2a (1.808 ± 0.10).

*A*/*C*_*i*_ curves showed a significant increase in photosynthetic rate (*A*) correlating with raising intracellular CO₂ concentration (*Ci*) in all genotypes (Fig. 4e). However, Su_*epfl9*-2a showed an increase in *A* comparable to Su_WT, while Su_*epfl9*-2b showed a more gradual response, leading to significantly lower *A* compared to WT across all *Ci* points (*P* < 0.05) (Fig. 4e). The maximum electron transport rate (*J*_max_) and the maximum Rubisco carboxylation rate (*V*_cmax_) were slightly less in Su_*epfl9*-2b than in Su_WT and Su_*epfl9*-2a, but not statistically different (respectively *P* = 0.12 and *P* = 0.299) (Fig. 4f and 4g).

### Reduced leaf transpiration and higher VPD breakpoints are associated with the reduction in stomatal density induced by *VviEPFL9*-2 knock-out

**Fig. 5:**
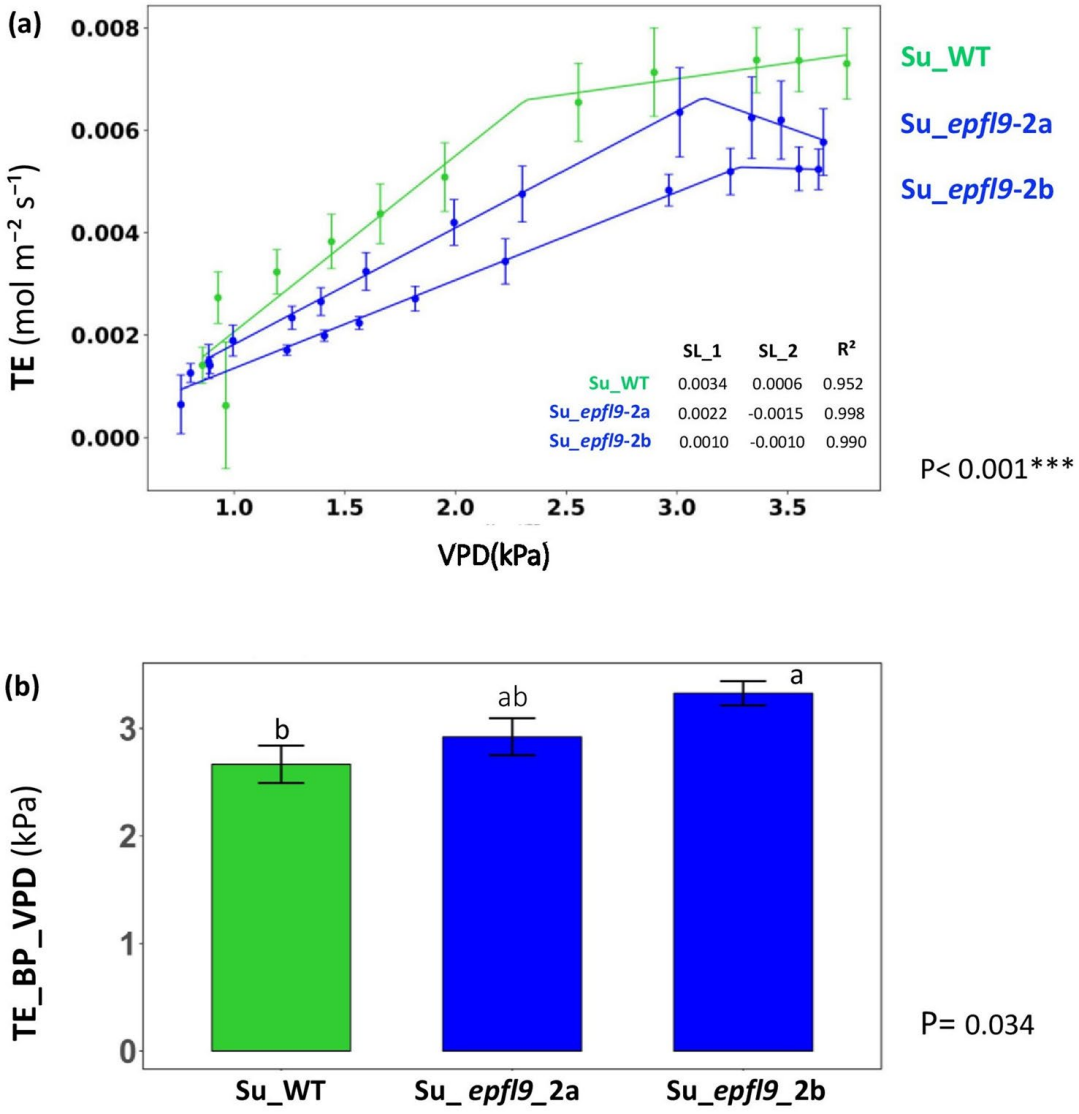
Transpiration rate in *epfl9*-2 mutants and WT under increasing VPD. **a** The transpiration rate (TE) was measured in relation to increasing vapor pressure deficit (VPD) levels, which were adjusted from 0.5 to 4 kPa. SL1 = the slope of the first linear segment; SL2 = the slope of the second linear segment; R2 coefficient of determination. **b** The VPD breakpoint (BP). Bars represent the mean ± SE (n = 5). Statistical significance was determined by one-way ANOVA followed by Tukey's post-hoc test (*P* < 0.05)

As shown in Fig. 5a, plant transpiration (TE) increased linearly with increasing vapor pressure deficit (VPD) in all genotypes, but with a progressively lower slope from Su_WT, to Su_*epfl9*-2a and finally to Su_*epfl9*-2b, consistent with a lower stomatal density phenotype. As the relative humidity of the air decreases, the critical reduction in transpiration rate was reached at higher VPD values in mutants lines compared to WT, with VPD breakpoints of 2.31 ± 0.32 kPa, 3.11 ± 0.04 kPa and 3.28 ± 0.47 kPa, respectively for Su_WT, Su_*epfl9*-2a and *epfl9*-2b. Statistical analysis showed a significant difference between Su_WT and Su_*epfl9*-2b (*P* < 0.001), while Su_*epfl9*-2a, as already reported for the photosynthetic response, had an intermediate behavior. A reduction in transpiration as a result of lower stomatal density was expected. However, it is interesting to note that the mutant plants reached a critical water status, implying the progressive stomatal closure, at higher VPD values (VPD breakpoints) compared to the WT.

### *VviEPFL9*-2 knock-out may improve water saving behavior under water stress conditions

**Fig. 6:**
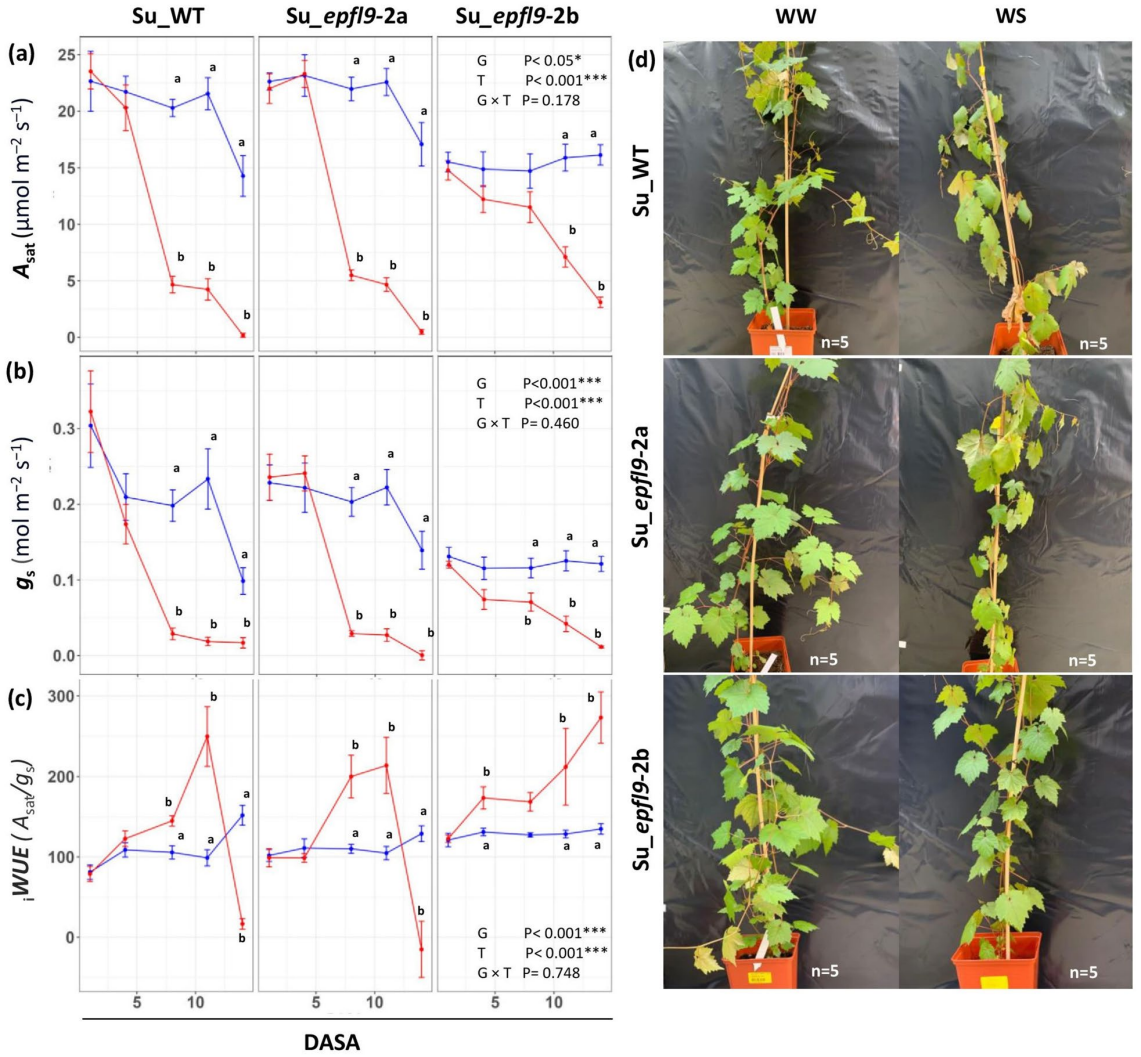
Photosynthetic traits in *epfl9*-2 mutants and WT under well-watered (WW) and water stress conditions (WS) (Experiment 1). **a** CO_2_ assimilation rate at saturating light (*A*_*sat*_), **b** stomatal conductance (*g*_*s*_), and **c** intrinsic water-use efficiency (*iWUE*) calculated as *A*_*sat*_*/g*_*s*_ over a 14-day period under WW (blue line) and WS (red line) conditions. Statistical significance was determined by one-way ANOVA followed by Tukey's post-hoc test (*P* < 0.05, *n* = 5). DASA = Days After Stress Application. **d** Images of the plants after the 14-day period

As shown in Fig. 6 (Experiment 1), the CO_2_ assimilation rate (*A*_sat_) exhibited a significant decline under water stress (WS) conditions across all genotypes. In the Su_WT, photosynthesis decreased from 20.29 ± 4.51 µmol m⁻^2^ s⁻^1^ to approximately 4.66 ± 1.64 µmol m⁻^2^ s⁻^1^ by the eighth day after the start of stress application (DASA). A similar trend was observed in the Su_*epfl9*-2a line, where the rate of photosynthesis also dropped from 23.29 ± 2.67 µmol m⁻^2^ s⁻^1^ to nearly 5.49 ± 1.06 µmol m⁻^2^ s⁻^1^ within the same period. Conversely, the mutant line Su_*epfl9*-2b, despite exhibiting a significantly lower CO_2_ assimilation rate under well-water (WW) conditions compared to WT (about 15 µmol m⁻^2^s⁻^1^ for Su_*epfl9*-2b vs about 20 µmol m⁻^2^s⁻^1^ for Su_WT), showed a more moderate *A* reduction during water stress, from 14.89 ± 2.65 µmol m⁻^2^s⁻^1^ to 11.51 ± 3.04 µmol m⁻^2^s⁻^1^ by the eighth day after the start of the experiment (DASA), reaching an *A* value of 3.10 ± 1.02 µmol m⁻^2^s⁻^1^ on day 14, corresponding to the end of the stress treatment. Statistical analysis revealed significant effects for both genotype (*P* = 0.015) and treatment (*P* < 0.001), while the genotype-by-treatment interaction was not significant (*P* = 0.178) confirming a different behavior for Su_*epfl9*-2b compared to the Su_WT. Similar to *A*, *g*_s_ dropped markedly under water stress for all genotypes (Fig. 6b), although the impact was less pronounced for *epfl9*-2b. Wild-type plants showed a steep decline in *g*_s_, from an initial 0.32 ± 0.12 mmol m⁻^2^ s⁻^1^ to 0.03 ± 0.01 by the eight DASA. In contrast, the mutant lines, while displaying lower *g*_s_ values in comparison to WT at the outset of the experiment and in WW conditions (in accordance with the observed SD), maintained higher *g*_s_ values over the stress progression, particularly Su_*epfl9*-2b. Statistical analysis indicates a significant effect of genotype (*P* = 0.001) and treatment (*P* < 0.001) on *g*_s_, while the interaction between genotype and treatment was not statistically significant (*P* = 0.46). As expected, intrinsic water use efficiency (_i_*WUE*), calculated as the ratio of assimilation (*A*) to stomatal conductance (*g*_s_) (Fig. 6c), increased sharply for all the genotypes under water stress (WS), suggesting a transient increase in efficiency due to reduced stomatal conductance combined with relatively higher CO_2_ assimilation rate early in the stress period. However, for mutant lines higher _i_*WUE* were recorded along stress application, the best performing resulting Su_*epfl9*-2b whose _i_*WUE* increased constantly along the experimental period. Under WW conditions, _i_*WUE* remained relatively constant across all genotypes. Statistical analysis showed significant effects of genotype (*P* < 0.001) and treatment (*P* < 0.001) on _i_*WUE*, while the genotype-by-treatment interaction was not significant (*P* = 0.43).

In addition, the water-stressed Su_WT exhibited a marked decline in stem water potential reaching values as low as -1.4 MPa, which indicates severe water stress. On the contrary, the Su_*epfl9*-2b mutant line maintained significantly higher stem water potential under WS condition with Ψstem values close to -0.4 MPa. This suggests the activation of a drought tolerance mechanism in Su_*epfl9*-2b. Consistently with gas exchange data, the mutant line Su_*epfl9*-2a showed a similar stressful state as WT under water deprivation, albeit with an intermediate degree between the wild type and Su_*epfl9*-2b (Fig. **S4**).

**Fig. 7:**
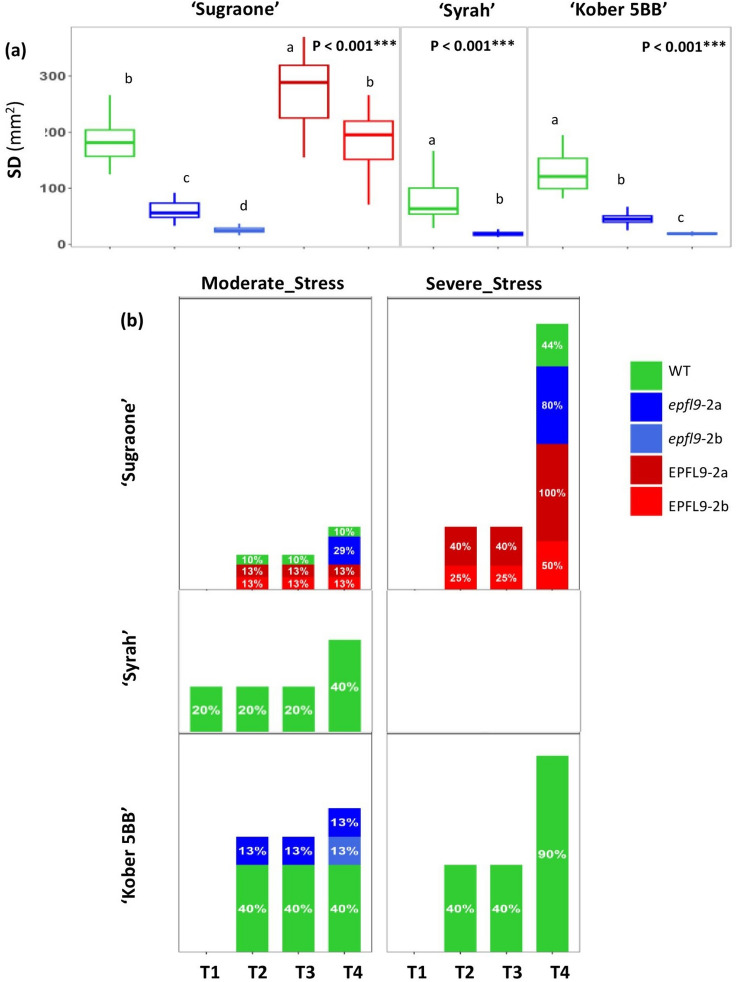
Mortality rate in modified lines for *VviEPFL9*-2 and WT under water stress conditions (Experiment 2). **a** Stomatal density evaluated for the plants used in Experiment 2: WT, *epfl9*-2a and *epfl9*-2b edited lines of different varieties (‘Sugraone, ‘Syrah, and ‘Kober 5BB’) and *VviEPFL9*-2 ‘Sugraone’ overexpressing lines (EPFL9-2a and EPFL9-2b). For each line and WT, 5 biological replicates were analysed, and, for each biological replicate, one fully expanded leaf. Statistical significance was determined by one-way ANOVA followed by Tukey's post-hoc test (*P* < 0.05). **b** Mortality rate (% of dead plants out of the total number of plants exposed to a stress condition), respectively for ‘Sugraone’, ‘Syrah’, ‘Kober 5BB’. For each line, the number of biological replicates subjected to different water conditions is reported in Table S5

In experiment 2 (Fig. 7, Fig. **S5,S6** and Table **S5**), in addition to ‘Sugraone’, edited plants of other genotypes were included, ‘Syrah’ and ‘Kober 5BB’, together with two ‘Sugraone’ EPFL9-2 overexpressing (OE) lines, Su_EPFL9-2a and Su_EPFL9-2b, which showed a *VviEPFL9*-2 transcript fold increase of 126 and 10.6 compared to Su_WT, respectively. In terms of plant growth, focusing on the ‘Sugraone’ cultivar, in control irrigation conditions the edited lines Su_*epfl9*-2a and Su_*epfl9*-2b exhibited a behavior that was, respectively, very similar and significantly different from the WT: the growth rate of Su*_epfl9*-2b was initially slower than that of the WT and Su_*epfl9*-2a, but it increased more during the 30-day observation period (Fig. **S5**). In conditions of water stress, the growth of all ‘Sugraone’ plants was severely reduced. Notably, in severe stress conditions, the Su_WT exhibited the most negative performance, which was also due to the death of several biological replicates. Regarding ‘Sugraone’ EPFL9-2 OE lines (Su_EPFL9-2a exhibiting a significantly higher SD than WT while Su_EPFL9-2b showing no significant SD deviation from the WT, Fig. 7a), they showed a growth comparable to the WT (Fig. **S5**). For the ‘Kober 5BB’ genotype, in conditions of good irrigation the initial growth of the WT plants was greater than that of the edited ones, then it remained constant during the 30-day observation period. However, under conditions of moderate stress, the growth rate was very low for both the WT and the edited lines while in conditions of more severe stress, the worst performance was shown for the WT, as several biological replicates died. The results obtained for the cv. ‘Syrah’ were found to contrast with those obtained for 'Sugraone' and 'Kober 5BB', since the WT plants exhibited lower growth in comparison to the edited plants under all three irrigation conditions. It is interesting to note that even under severe water stress, the growth rate was positive. In addition to plant growth, mortality data were also collected. As shown in Fig. 7b the mortality rate was, in general, higher for the WT plants compared to the edited ones across all three genotypes. 'Kober 5BB' was the most affected genotype, with a high mortality rate already at 14 days after moderate stress initiation, followed by 'Sugraone'. Under moderate stress conditions, Su_*epfl9*-2a exhibited a delayed onset of death compared to WT, whereas in severe stress its mortality rate was found to exceed that of WT, thereby adding new evidence that this line and WT have similar physiological characteristics. Conversely, it is interesting to note that the mortality rate of ‘Sugraone’ plants overexpressing the *VviEPFL9*-2 gene was the highest.

## Discussion

Extreme weather conditions, such as rising temperatures, high VPD levels, and soil drought, are likely to impact the future of agriculture (Rivero et al. 2022) and in general woody-plant mortality (McDowell et al. 2022). One of the natural physiological adaptations that many plant species adopt to cope with prolonged periods of water stress is a reduction in stomatal density (Chater et al. 2014). Stomata, the entry gates for CO_2_, which account for 90–95% of water loss under natural environmental conditions (Jones 1998), constitute a key hub for the fundamental plant processes of transpiration and photosynthesis. A better understanding of the genetic regulation that underlies their function in each species is therefore crucial, also to identify target genes for the genetic improvement of crops in order to mitigate abiotic stresses exacerbated by climate change (Nerva et al. 2023).

Our work showed for the first time the key role of *VviEPFL9*-2 over *VviEPFL9*-1 in determining stomatal density in mature grapevine leaves by comparing *epfl9*-1 and *epfl9*-2 mutants of the same age and similar size, thus demonstrating the functional divergence of the *EPFL9* paralogs in grapevine. A different function of the two paralogs was also suggested by their expression profile, which was assessed in both greenhouse and field experiments (Fig. 1), as well as by data obtained from publicly available RNAseq datasets (Fig. 1g). The observation that *VviEPFL9*-1 is mainly expressed in leaf primordia of the shoot tip, whereas *VviEPFL9*-2 is expressed in leaf primordia as well as in leaves at different stages (with a peak near full maturity), suggests that both genes may be involved in fixing the maximum number of stomata for the leaf primordia, while only *VviEPFL9*-2 exerts a further control for stomatal density during leaf expansion. A similar picture was observed in rice, where the transcript abundance of the two *EPFL9* paralogs differed in leaf tissues, with one greatly exceeding the other (Karavolias et al. 2023). Furthermore, these authors found that the expression of both genes was minimal in adult leaves (Karavolias et al. 2023). This is in line with what was found in poplar (Hamanishi et al. 2012), where fully developed leaves showed a stomatal set that did not change even after a period of water deficit. In contrast, leaves developing under water deficit had a lower stomatal density and index compared to leaves of the same stage developing under well-watered conditions. In the same study it has been shown that *EPFL9* is among the set of genes whose expression is modulated in response to drought (Hamanishi et al. 2012). Consistent with this finding, we observed that the expression of *VviEPFL9*-2 in grapevine leaves is significantly modulated by the exogenous application of abscisic acid (ABA), the hormone that accumulates under water stress (Fig. 2), suggesting a mechanism of repression of *VviEPFL9*-2 induced by water deficit conditions. It is noteworthy that the dynamics of the response of *VviEPFL9*-2 expression to ABA were consistent across both 'Cabernet Sauvignon' and 'Syrah' cultivars, exhibiting comparable patterns of repression and recovery. This indicates that the regulation of *VviEPFL9*-2 by ABA may represent a conserved response mechanism in grapevines. In *Arabidopsis*, it has been clearly demonstrated that ABA regulates stomatal formation by activating the kinase SnRK2, which in turn phosphorylates SPCH at specific residues, thereby connecting ABA/drought signals to stomatal suppression (Yang et al. 2022). Interestingly, our data point to the possibility that ABA may also destabilize SPCH via an alternative route, namely the one controlled by EPF-peptide. This hypothesis is further supported by the findings of Liu et al. (2016), who demonstrated that *PdEPF2*, the EPFL9 antagonistic peptide, was induced by ABA in poplar. In addition, Zhao and colleagues (2022), in apple, observed that the transcription factor *MdHB7*-like is upregulated in response to ABA treatment and its overexpression leads to a reduction of *MdEPFL9.1* and *MdEPFL9.2* transcripts, whereas its silencing via RNAi leads to *MdEPFL9.1* and *MdEPFL9.2* up-regulation, thereby indicating a connection between ABA and EPFL9 (Zhao et al. 2022). The intervention of intermediate transcription factors, potentially stage-specific, in the link between ABA and *VviEPFL9*-2, could explain the differential regulation of this gene in leaves and leaf primordia, where the repression effect is observed (Fig. 2c, f) or not (Fig. 2b, e), respectively.

Reducing stomatal density (SD) is expected to improve drought tolerance in plants by limiting plant water loss through transpiration (Hepworth et al. 2015; Hughes et al. 2017; Jiao et al. 2022). Stomatal density controls stomatal conductance (*g*_s_) and transpiration per unit of leaf area (Murray et al. 2019; Sun et al. 2023) and it is a critical factor for mesophyll photosynthesis (Lawson et al. 2014). In fact, a reduction in stomatal density may not only affect transpiration, but also photosynthesis, with negative effects on plant growth, fruit quality and yield (Rodrigues et al. 2019a; Karavolias et al. 2023). In this study, the *epfl9*-2a and the *epfl9*-2b mutant lines, with 61 and 84% lower SD than Su_WT, respectively, were subject to an extensive physiological evaluation under both well-watered and water-stressed conditions. Despite a similar reduction in stomatal density, the mutated lines showed different physiological responses. As shown by our analysis in the greenhouse, *epfl9*-2a maintained *A*,* g*_s_ and _i_*WUE* comparable to the wild type (WT) while these parameters were significantly different between *epfl9*-2b and WT (Fig. 4a,b,c). An intermediate photosynthetic response of *epfl9*-2a, in many cases significantly different from *epfl9*-2b but not from Su_WT, was also observed when light and CO_2_ curves were performed and during the water stress experiments (Fig. 4d,e, 6, 7). Furthermore, this intermediate behavior was confirmed by dark respiration and by the transpiration assay with increasing air VPD levels (Fig. 5).

The response of *epfl9*-2b is somewhat predictable, as a significant reduction in stomatal density may reduce CO₂ uptake, thereby limiting photosynthetic capacity, while allowing longer conservation of plant water pools, which is beneficial under conditions of water scarcity (Lawson et al. 2014). This is due to the tight trade-off between water use efficiency and photosynthetic performance, determined by stomatal dynamics (Faralli et al. 2019). In contrast, the response of *epfl9*-a, which does not seem to be affected by a strong reduction in SD in terms of photosynthesis, is more enigmatic. The underlying reason may be ascribed to multifaceted aspects including stomatal morphological characteristics (SD and stomatal size) and movement dynamics (opening and closing) as well as to CO_2_ mesophyll conductance (g_m_). Moreover, a possible role for vascular hydraulic conductance in this equilibrium is not to be excluded. Regarding the morphological factor, it has been found in several studies (Franks et al. 2009; Doheny-Adams et al. 2012; Lawson & McElwain 2016) that stomatal density (SD) and stomatal size (SS) have a negative but non-linear correlation for optimizing the use of epidermal space as an adaptive strategy. However, no significant increase in stomatal pore size was detected for Su_*epfl9-*2a compared to Su_WT (Fig. 3d). Similar findings were observed in rice, barley and sugarcane, where a decrease in SD was not accompanied by a change in SS (Hughes et al. 2017; Schuler et al. 2018; Lunn et al. 2024). Furthermore, improved mesophyll conductance (*g*_m_) with enhanced internal CO_2_ diffusion may be a compensatory mechanism capable of mitigating the effects of lower SD (Flexas et al. 2013; Lundgren & Fleming 2020). A strong relationship has been found between stomatal distribution and mesophyll airspace (Lundgren et al. 2019), which is thought to be developmentally regulated and evolutionarily selected (Baillie and Fleming 2020; Lundgren et al. 2019). In grapevine, a positive correlation between leaf mesophyll porosity and mesophyll conductance has been observed (Tomás et al. 2014). Finally, since a strict association between photosynthetic and hydraulic traits has been extensively shown (Zhu et al. 2018; Deans et al. 2020), it may be speculated that a modulation of vascular traits in Su_*epfl9*-2a has had an influence on stomatal conductance and photosynthesis. Indeed, the relationship between photosynthesis and vessel hydraulics may be extrapolated by the characterization of the ‘Sugraone’ edited plants at increasing VPD values (Fig. 5). These results indicate that edited lines, Su_*epfl9-*2b more than Su_*epfl9*-2a, maintained stomatal opening even at high VPD levels, at which transpiration has already started to decrease in WT plants. Furthermore, the VPD threshold (VPD Breakpoints), which denotes the critical point after which stomata partially close to induce stomatal limitation of transpiration, was observed to be higher in edited plants than in the WT. High-VPD-breakpoint trait can be beneficial for the plant in order to maintain stable photosynthesis and evaporative cooling capacity for periods of low water availability, at least under Mediterranean environmental conditions where intermittent rainfall is expected. Previous studies suggest that the limited growth and high mortality associated with drought derives from water and carbon depletion and from a decline of their flows which are no longer able to satisfy the demands of the plant tissues (McDowell et al. 2022) as well as from hydraulic failure (Grossiord et al. 2020). A recent study in grapevine showed that in water-stressed plants, starch depletion in the stem was more severe in the phloem than in the xylem ray parenchyma, resulting in the death of metabolically active cells in the phloem (Prats et al. 2023).

The divergent behavior exhibited by the two edited lines of the ‘Sugraone’ variety led us to extend the evaluation to other genotypes in an additional water stress experiment (Exp. 2) in order to obtain a broader dataset (a total of 254 plants were used, Table S5) from which to draw more general conclusions. Moreover, it’s worth to highlight that the two water stress experiments differed not only for the parameters evaluated (gas-exchange in the first experiment and plant growth and mortality rate in the second one) but also in terms of duration and environmental factors (the first experiment was conducted in early spring under stable conditions of temperature and humidity, while the second was conducted in summer without a control of the RH and temperature, which on some days exceeded 40 °C as indicated in Fig. **S6**). Significant variations in the growth rate of the edited and WT plants across different genotypes were observed indicating a potential genotype-dependent effect. For the cultivars 'Sugraone' and 'Kober 5BB', *epfl9*-2 edited plants exhibited reduced or comparable growth compared to WT under well-watered and water-stress conditions, while the 'Syrah' *epfl9*-2 edited plant demonstrated enhanced growth than WT. Moreover, ‘Syrah’, which is an anisohydric cultivar (Faralli et al. 2022; Herrera et al. 2022) showed the highest survival rate to water stress with zero mortality registered for *epfl9*-2 edited plant. These findings support the conclusion that a significant reduction in stomatal density may have a variable impact on the photosynthetic process of the plant and as a consequence on plant growth, the regulation of which may be orchestrated by a multitude of metabolic pathways and physiological dynamics at the cellular and tissue level, capable of compensating for any induced imbalance. In contrast, the reduction in stomatal density has been demonstrated to be pivotal in enhancing plant survival under water stress conditions, irrespective of cultivar. This finding is corroborated by the poorest survival rate exhibited by the EPFL9-2 overexpressing lines, which have the highest SD.

Although grapevine is a woody fruit crop well adapted to the warm and dry Mediterranean climate (Costa et al. 2023), climate change is a significant challenge for many viticultural regions of the world (van Leeuwen et al. 2024). Many studies have investigated the possible impacts of a warmer and drier environment on viticulture in the coming decades (Costa et al. 2019; Gambetta et al. 2020; Faralli et al. 2024), and numerous actions have been implemented to provide solutions and thus preserve the high economic, societal, and cultural value of grapevine (Gambetta et al. 2020). Provisional models have been developed to identify viticultural regions at higher risk of water stress (Santillán et al. 2020; Hofmann et al. 2022; Zito et al. 2023; Prada et al. 2024), and more adapted varieties and rootstocks are being selected (Lamarque et al. 2023). In our study we showed that the reduction in stomatal density resulting from the knockout of *VviEPFL9*-2 enhances plant water use efficiency and resilience to water stress in grapevine. Modification of this key gene in cultivars of interest using advanced new genomic techniques, such as DNA-free editing of protoplasts (Scintilla et al. 2022), could serve as a tool to adapt grapevine genetics to future climatic scenarios and to maintain internationally and locally appreciated cultivars in traditional wine-growing regions.

## Supplementary Information

Below is the link to the electronic supplementary material.

Supplementary file1 (DOCX 1802 KB)

Supplementary file2 (XLSX 1812 KB)

Supplementary file3 (XLSX 87 KB) Fig. S1 Area of leaves used for VviEPFL9-1 and VviEPFL9-2 expression analysis (Fig. 1). Fig. S2 Qualitative PCR check of ‘Sugraone’ epfl9-1 and epfl9-2 mutant lines independency. Fig. S3 Allele plot of the target site in loss-of-function mutants of ‘Syrah’ (Sy) and ‘Kober 5BB’ (Ko) according to the analysis of the Illumina sequencing by CRISPResso2. Fig. S4 Stem water potential of ‘Sugraone’ plants subjected to water stress experiment 1. Fig. S5 Plant height during water stress experiment 2. Fig. S6 Temperatures and irrigation schedules of water stress experiment 2. Table S1 Primers used in the PCR reactions for different applications. Table S2 Metadata associated with the transcriptomic public Illumina-based transcriptomic runs reanalysed and graphed in Fig. 1g.Table S3 Metadata associated with the transcriptomic public Illumina-based transcriptomic runs reanalysed and graphed in Fig. 2g,h. Table S4 Molecular characterization of edited plants of Vitis vinifera cv. ‘Sugraone’ and ‘Syrah’ and of the rootstock ‘Kober 5BB’ (Vitis berlandieri x Vitis riparia). Table S5 Plants used in water stress experiment 2

## Data Availability

The original contributions presented in the study are included in the article/Supporting information and further inquiries can be directed to the corresponding author/s.
