# Supplementary material for: Reduced stomatal density improves water-use efficiency in grapevine under climate scenarios of decreased water availability
Source: Plant Cell Rep. 2025 Aug 7;44(9):195. doi: 10.1007/s00299-025-03577-9 (PMC12331838; doi:10.1007/s00299-025-03577-9)
Supplement: Supplementary file 1 — Supplementary file1 (DOCX 1802 KB) [file 299_2025_3577_MOESM1_ESM.docx]

**Supporting Information**

Article Title:

**Reduced Stomatal Density Improves Water-Use Efficiency in Grapevine Under Future Climate Scenarios of Decreased Water Availability**

**Plant Cell Reports**

Umar Shahbaz^1,2^, Pierre Videau^3^, Emma Coulonnier^3^, Carla Papon^3^, David Navarro-Payá^4^, Alvaro Vidal^1,2,4^, José Tomás Matus^4^, Mickael Malnoy^1^, Olivier Zekri^3^, Fabio Fiorani^5^, Michele Faralli^2^, Lorenza Dalla Costa^1^*

Author for correspondence: Lorenza Dalla Costa

e-mail: lorenza.dallacosta@fmach.it

ORCID: <https://orcid.org/0000-0001-5915-5582>

Research and Innovation Centre, Fondazione Edmund Mach, San Michele all’Adige, Italy


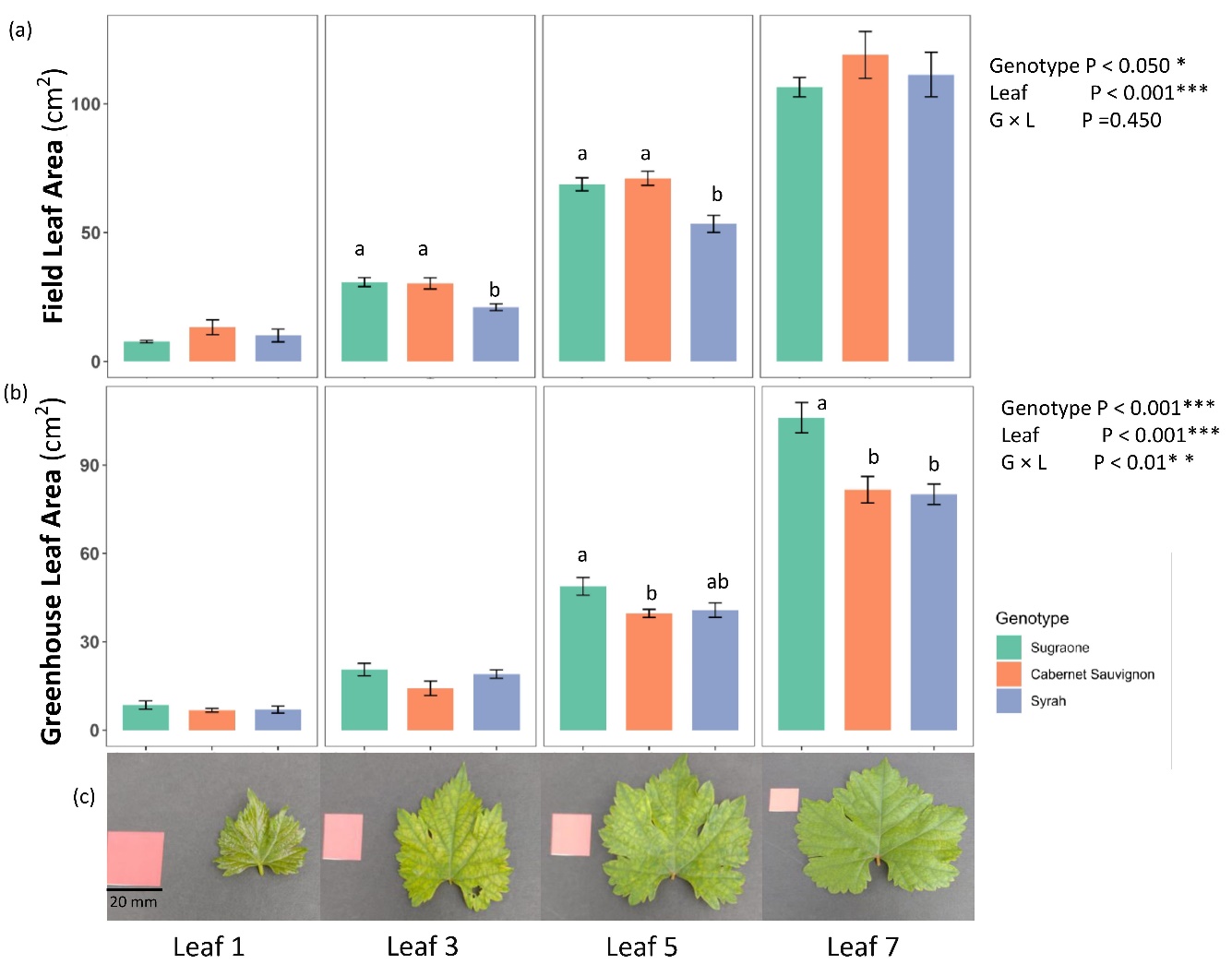


**Fig. S1** Area of leaves used for *VviEPFL9*-1 and *VviEPFL9*-2 expression analysis (Fig. 1). **(a)** Area of leaves from field plants **(b)** Area of leaves from greenhouse plants **(c)** Images taken by phone cell and analyzed with the open-source application “Easy leaf area” (https://www.quantitative-plant.org/software/easy-leaf-area) that uses the RGB value of each pixel to identify leaf and scale regions in each image. The red square in each image measures 2 x 2 cm^2^.

*epfl9*-2 ‘Sugraone’ lines

*epfl9*-1 ‘Sugraone’ lines

**
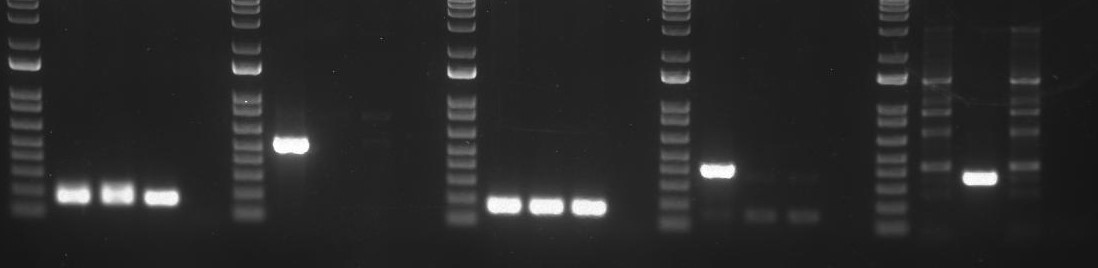
**

**Endogenous gene (165 bp)**

**1a 1b SuWT c-**

**Chr. 1_4310437 (509 bp)**

**1a 1b SuWT c-**

**Endogenous gene (165 bp)**

**2a 2b SuWT c-**

**Chr. 19_8696478 (404 bp)**

**2a 2b SuWT c-**

**Chr. 6_13603211 (363 bp)**

**2a 2b SuWT c-**

**L**

**L**

**L**

**L**

**L**

1.5 Kbp

1 Kbp

850 bp Kbp

400 bp

500 bp

650 bp

200 bp

300 bp

100 bp

**Fig. S2** Qualitative PCR check of ‘Sugraone’ *epfl9-*1 and *epfl9*-2 mutant lines independency. Primers in column 5 and 6 of Table S4 were used. L= Ladder 1KbPlus (Invitrogen). 1a= Su_*epfl9*-1a; 1b= Su_*epfl9*-1b; 2a= Su_*epfl9*-2a; 2b= Su_*epfl9*-2b.

| **Line Sy_*epfl9*-2a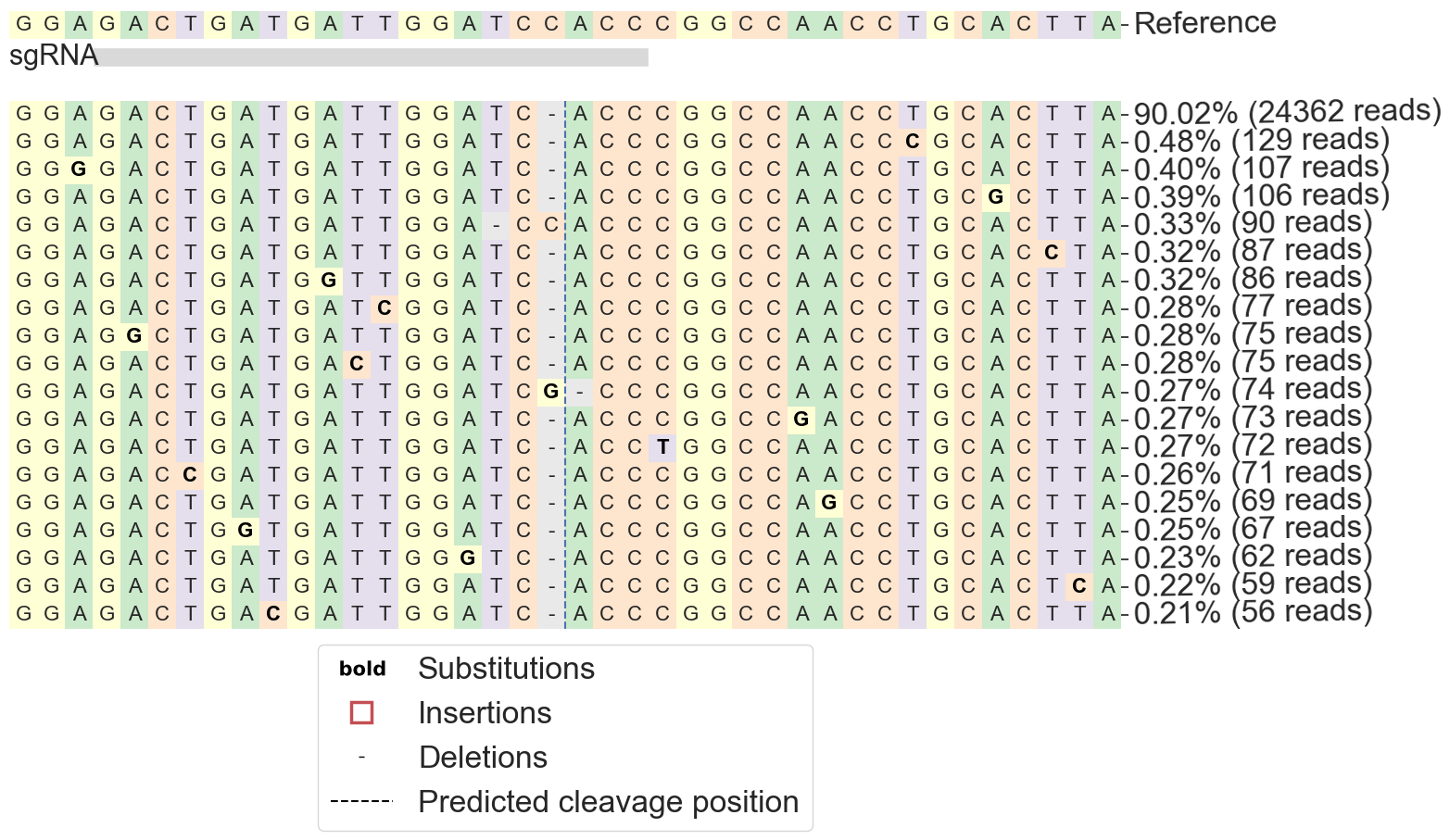** |
| --- |
|  |
| **Line Ko_*epfl9*-2a** |
| **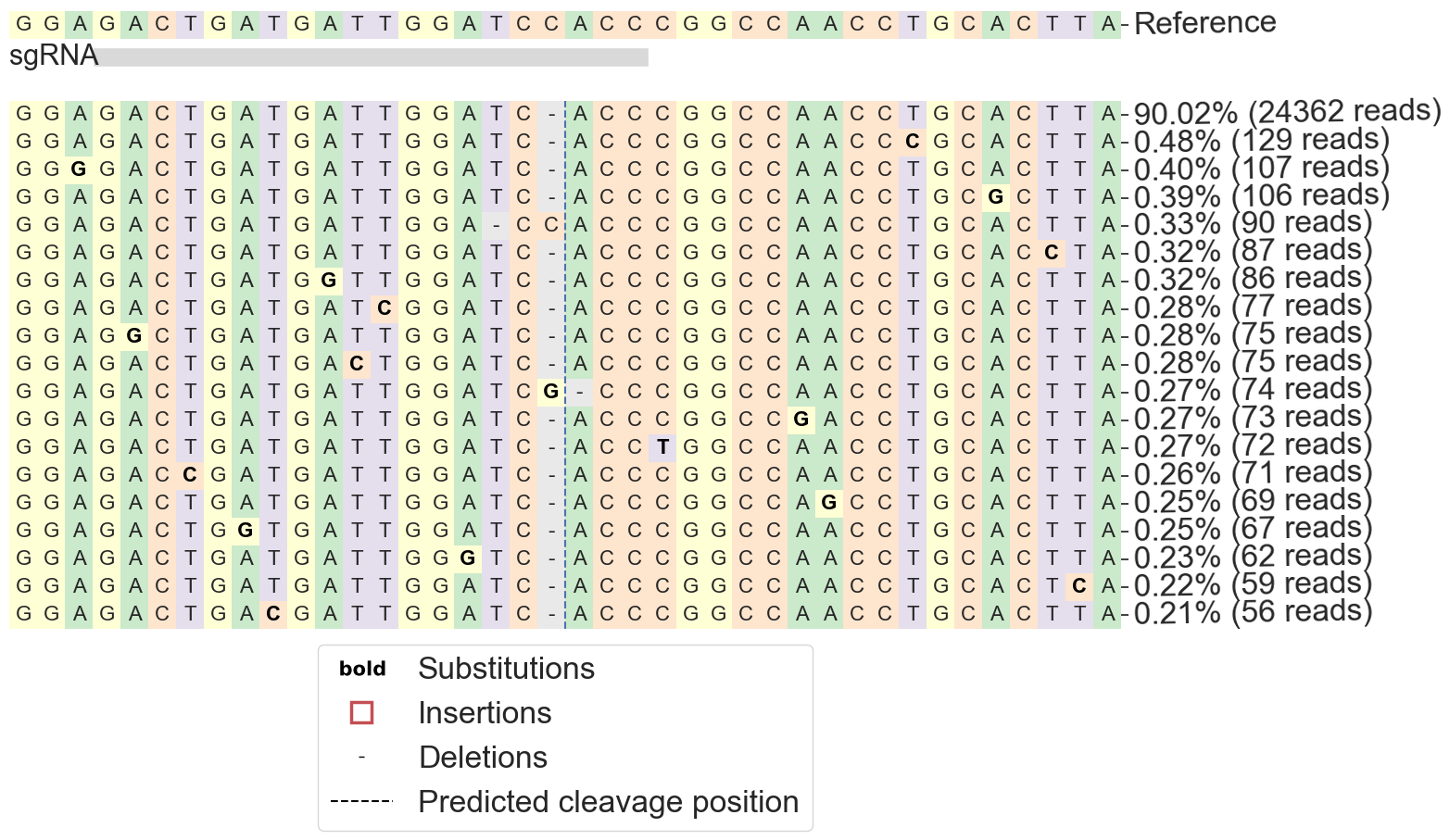**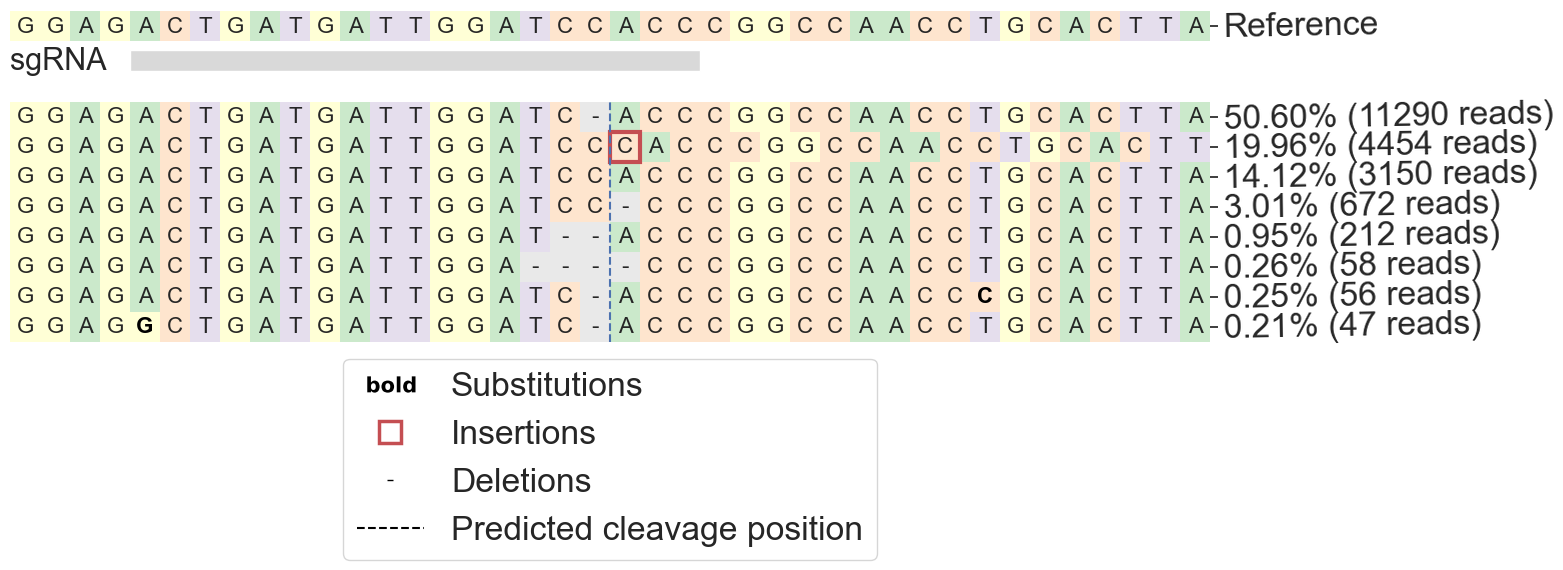 |
| **Line Ko_*epfl9*-2b** |
| 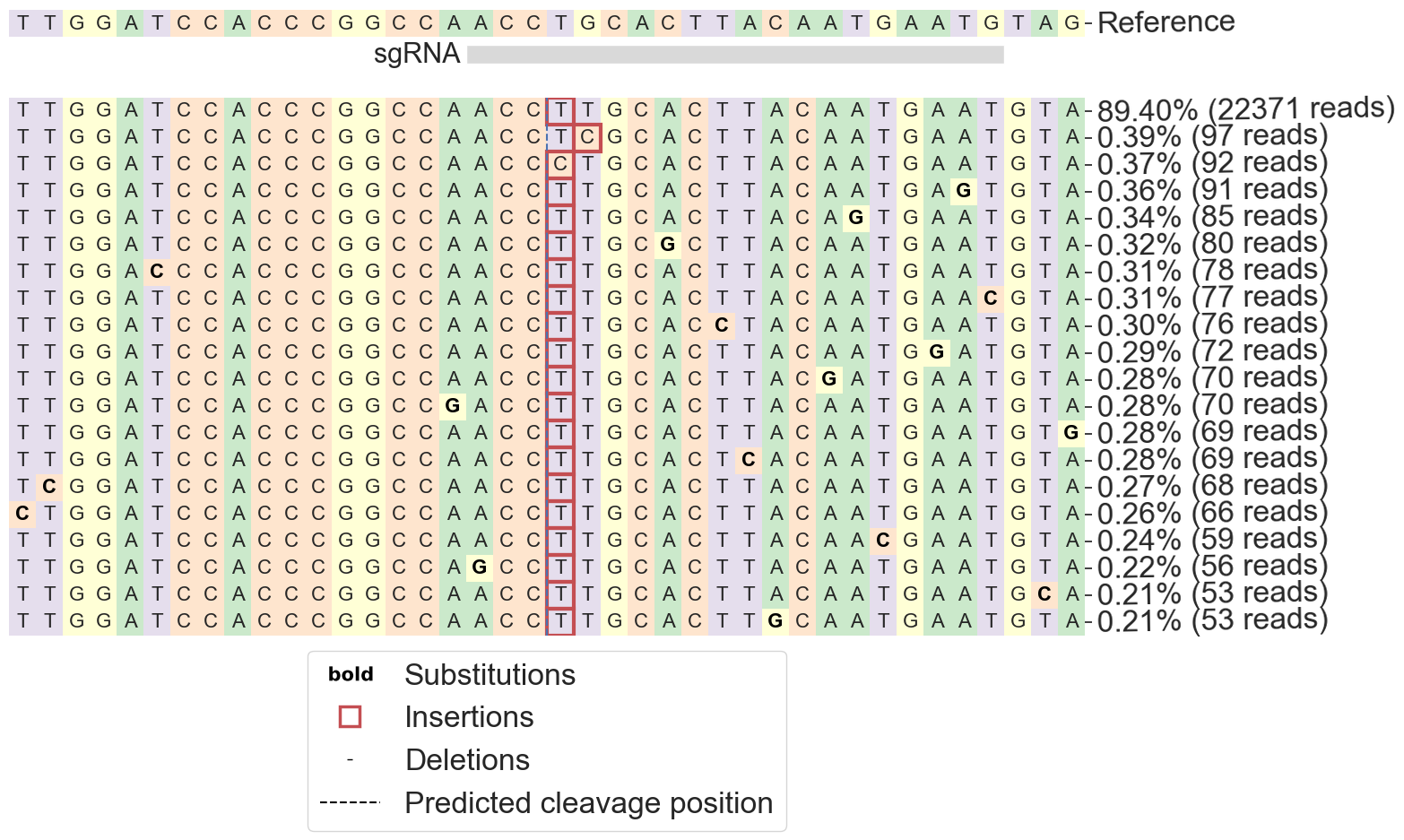 |

**Fig. S3** Allele plot of the target site in *loss-of-function* mutants of ‘Syrah’ (Sy) and ‘Kober 5BB’ (Ko) according to the analysis of the Illumina sequencing by CRISPResso2. The sgRNA was indicated with a grey line, sgRNA937 was used for line Ko_*epfl9*-2b while sgRNA938 was used for Sy_*epfl9*-2a and Ko_*epfl9*-2a lines.

**Su_WT Su-*epfl9*-2a Su-*epfl9*-2b**


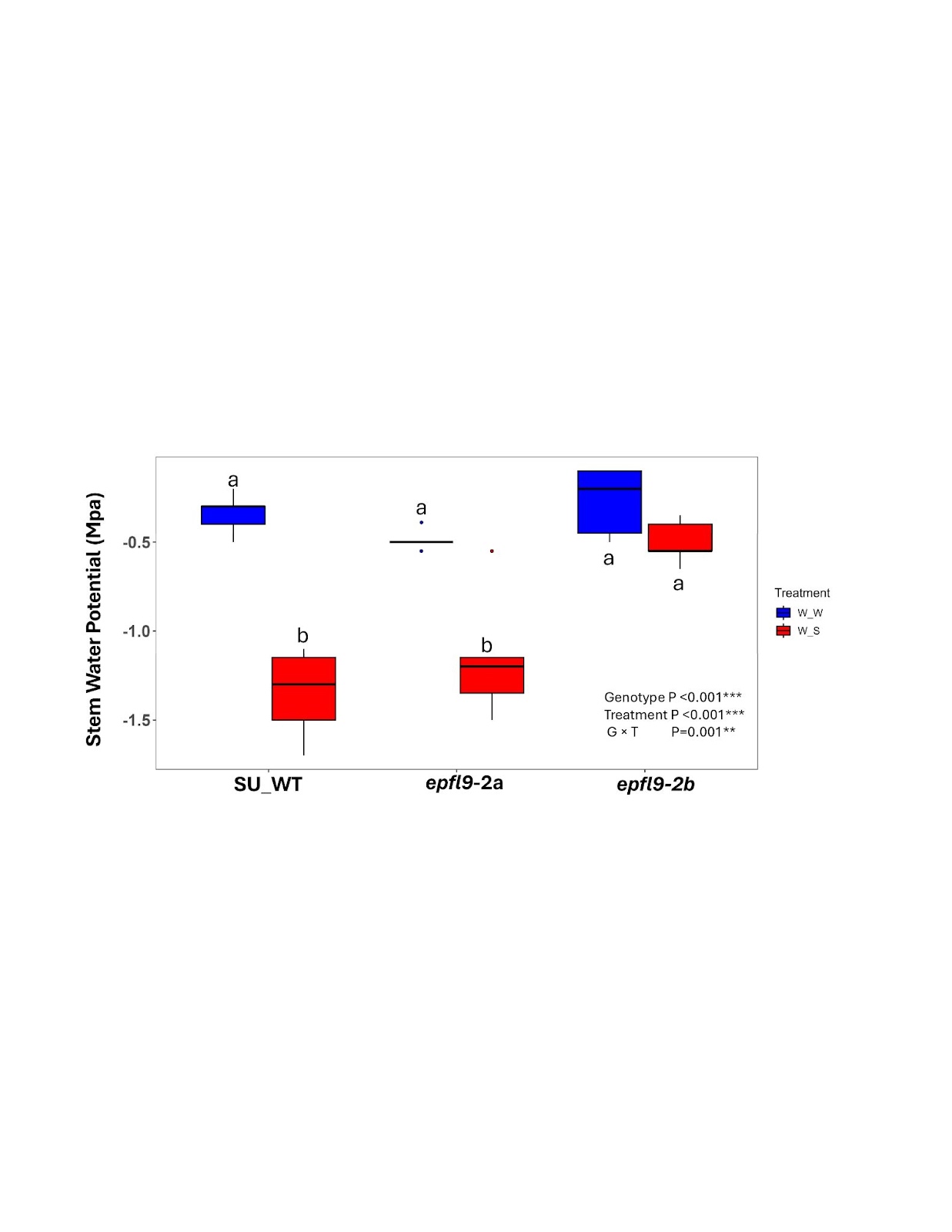


**Stem water potential (MPa)**

**Fig. S4** Stem water potential of ‘Sugraone’ plants subjected to water stress experiment 1. Bars represent the mean ± SE (n=5) of the stem water potential (MPa) values taken during midday on day 11 of the water stress experiment in the greenhouse under WW (blue) and WS (red) conditions. Statistical significance was determined by two-way ANOVA followed by Tukey's post-hoc test (P < 0.05).

**
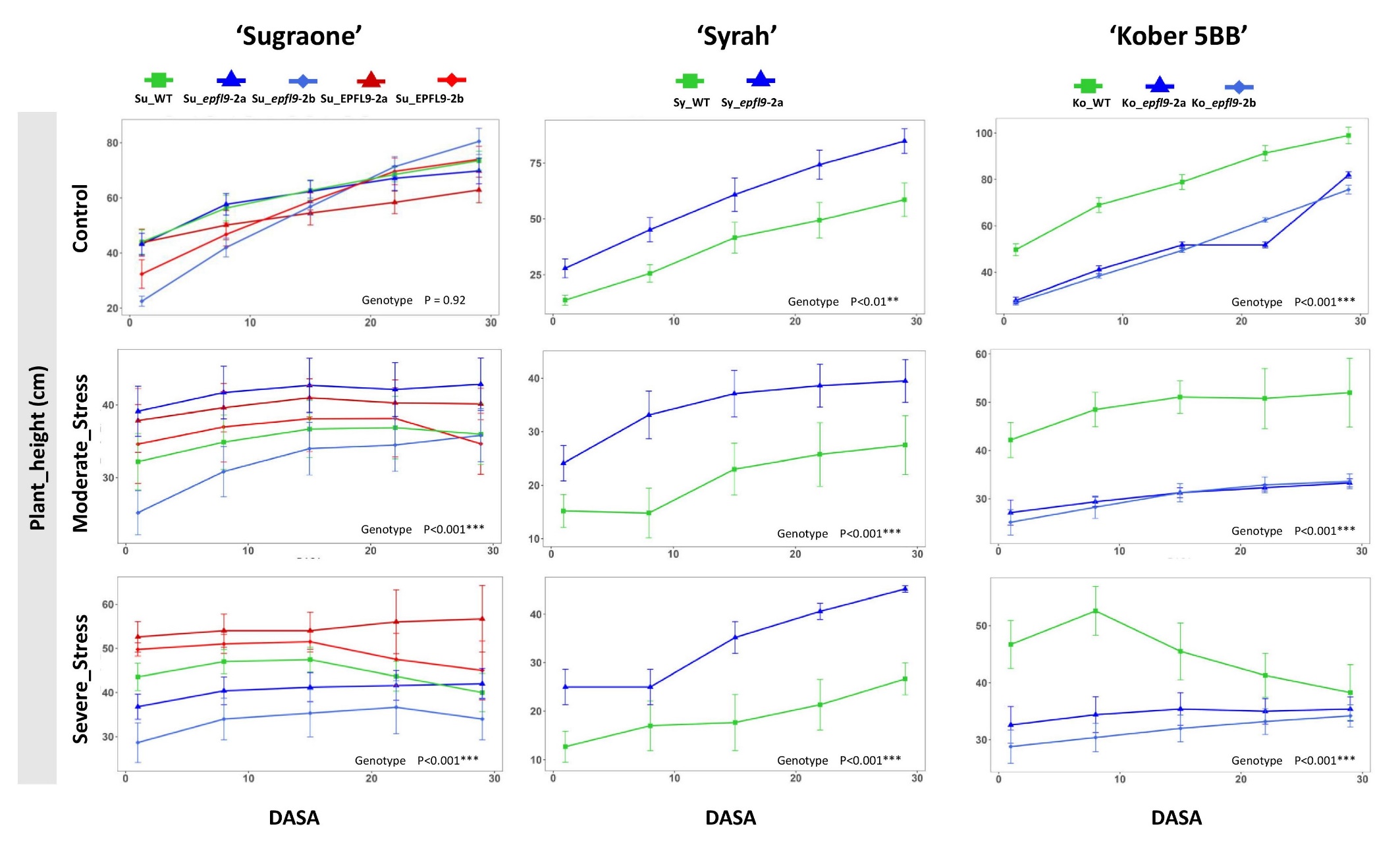
Fig. S5** Plant height during water stress experiment 2. Plant height (cm) was measured from the base of the stem to the apex in ‘Sugraone’ (WT, KO, and OE lines), ‘Syrah’ (WT and KO line), and ‘Kober 5BB’ (WT and KO lines) under control, moderate, and severe water stress. Measurements were taken weekly, starting from T0 (08/07/2024). All the plants were measured excluding those that died during the experiment.


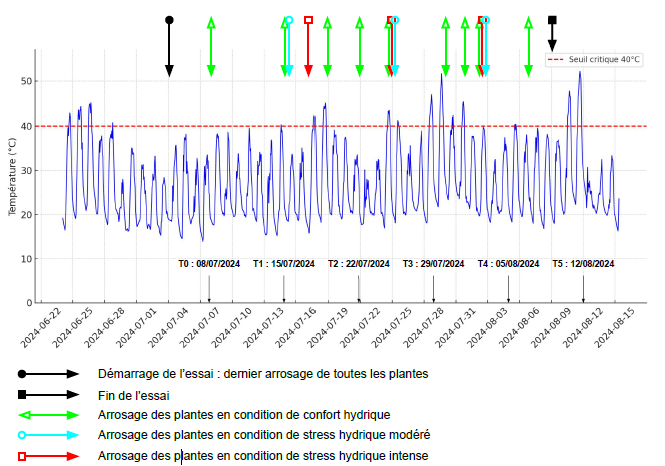


**Fig. S6** Temperatures and irrigation schedules of water stress experiment 2. The blue line illustrates greenhouse temperature variations during the experiment, while the red dashed line indicates the critical threshold of 40°C. Black arrows refer to the initiation and ending of the experiment, whereas green, blue, and red arrows indicate watering under control, moderate stress, and severe stress conditions, respectively.

| Primer name | Primer sequence | PCR product lenght | Application |
| --- | --- | --- | --- |
| SpCas9_Fw | 5′-CTTCAGAAAGGACTTCCAATTC-3′ | 693 bp | Screening of the transgenic edited lines |
| SpCas9_Rv | 5′-ATGATCAAGTCCTTCTTCACTT-3′ |  |  |
| nptII_Fw | 5’-GATGGATTGCACGCAGGTTC-3’ | 734 bp | Screening of the overexpressing lines |
| nptII_Rv | 5’-GGAGCGGCGATACCGTAAAG-3’ |  |  |
| VvEPFL9-1_San_fw | 5′-GGGTGCTAGAACAATGGAGATGG-3′ | 165 bp | Check of the editing in the target site (on/off target) with Sanger sequencing |
| VvEPFL9-1_San_rv | 5′-TCTCCTACATCCCACATGCATCT-3’ |  |  |
| OE-EPFL9_2-F | 5’-ATGCTCATCTCTAAACAACCC-3’ | 653 bp | Amplification of VviEPFL9-2 from genomic DNA for cloning in OE vector |
| OE-EPFL9_2-R | 5’-CCTATGACAAACGCATCTATAGT-3’ |  |  |
| VvEPFL9-1_ill_fw | 5′-GGGACTGCAACTCATTCAGAACT-3′ | 450 bp | Check of the editing in the target site (on/off target) with Illumina sequencing |
| VvEPFL9-1_ill_rv | 5′-TCTCCTACATCCCACATGCATCT-3’ |  |  |
| VvEPFL9-2_fw | 5′-GGGAACAAGTAGTATCTATGCCT-3′ | 308 bp | Check of the editing in the target site (on/off target) with Sanger and Illumina sequencing |
| VvEPFL9-2_rv | 5′- TGATCAACACAACACTGAGCCT-3′ |  |  |
| VvChiRT_fw | 5′-GAGGCTGGGGATGAGAAAATTG-3′ | 75 bp | CN quantification by Real-time PCR |
| VvChiRT_rv | 5′-CCCATCTCTCCTTCAACCACCT-3′ |  |  |
| SpCas9RT_fw | 5′-TACGCTGACCTTTTCTTGG-3’ | 87 bp |  |
| SpCas9RT_rv | 5′-CTTGGTGATCTCAGTGTTCA-3′ |  |  |
| VvEPFL9-1_Exp_fw | 5′-GGTCTACAGCCCCAACATGC-3′ | 74 bp | Gene expression by Real-time PCR |
| VvEPFL9-1_Exp_rv | 5′-AGGAATCTGCTCTGCTCTGC-3′ |  |  |
| VvEPFL9-2_Exp_fw | 5′-CGGCCAACCTGCACTTACAA-3’ | 112 bp |  |
| VvEPFL9-2_Exp_rv | 5′-CGCATCTATAGTGGTATGCGCT-3′ |  |  |
| ADAP_ill | 5′-***GTCTCGTGGGCTCGGAGATGTGTATAAGAGACAG***GTAATACGACTCACTATAGGGC-3’* | ^a^ | Integration site identification |
| P35S_ill | 5′-***TCGTCGGCAGCGTCAGATGTGTATAAGAGACAG***GCTGGGCAATGGAATCCGAG-3′* |  |  |

**Table S1** Primers used in the PCR reactions for different applications. ^a^ PCR product length is dependent from the random T-DNA insertion in the plant genome. *sequence in italics is the Illumina index adapter.

**Table S2** Metadata associated with the transcriptomic public Illumina-based transcriptomic runs reanalysed and graphed in Fig. **1g**.

Available in a separate file

**Table S3** Metadata associated with the transcriptomic public Illumina-based transcriptomic runs reanalysed and graphed in Fig. **2g,h**.

Available in a separate file

n.f.= not found

**Table S4** Molecular characterization of edited plants of *Vitis vinifera* cv. ‘Sugraone’ and ‘Syrah’ and of the rootstock ‘Kober 5BB’ (*Vitis berlandieri* x *Vitis riparia*). T-DNA CN (column 3) = copy number of T-DNA integration in the plant genome analyzed by the *Cas9* gene quantification via Real-time PCR (Dalla Costa et al., 2020). T-DNA position (column 4) = the genomic position of at least one T-DNA cassette identified via the NGS method (described in Dalla Costa et al., 2020) and validated on genomic DNA using the primers reported in columns 5 and 6.

| **Grapevine** | **Line ID** | **T-DNA CN** | **T-DNA position** | **Primers used to validate T-DNA integration point** | |
| --- | --- | --- | --- | --- | --- |
|  |  |  |  | annealing to T-DNA left side | annealing to genomic DNA |
| ‘Sugraone’ | Su_*epfl9*-1a | 2.70 ± 0.04 | Chr. 1; position 4310437 | Fw: 5′-GGGCAATGGAATCCGAGGA-3′ | Rv: 5′-ATATCCGCAGACACAGACGT-3′ |
|  | Su_*epfl9*-1b | 2.19 ±0.62 | n.f. | - | - |
|  | Su_*epfl9*-2a | 3.66 ± 0.3 | Chr. 19; position 8696478 | Fw: 5′-AACTTCGGCCGGTCTAGAGC-3′ | Rv: 5′-TGAGTCCAATCCAAGTAACACCA-3′ |
|  | Su_*epfl9*-2b | 3.86 ± 0.5 | Chr. 6; position 13603211 | Fw: 5′-AACTTCGGCCGGTCTAGAGC-3′ | Rv: 5′-GGGCTACTTGGAAGGGATGGG-3′ |
| ‘Syrah’ | Sy_*epfl9*-2a | 2.00 ± 0.52 | Chr. 13; position 3826701 |  |  |
| Kober 5BB | Ko_*epfl9*-2a | 0.97 ± 0.13 | Chr. 5; position 8090812 |  |  |
|  | Ko_*epfl9*-2b | 2.99 | Chr. 8; position 16964186 |  |  |

| **Genotype** | **Plant** | ***VviEPFL9*-2 modification** | **Well-water** | **Water-stress** | |
| --- | --- | --- | --- | --- | --- |
|  |  |  |  | **moderate** | **severe** |
| **‘Sugraone’** | WT |  | 10 | 10 | 9 |
|  | Su_*epfl9-*2a | KO | 9 | 7 | 5 |
|  | Su_*epfl9-*2b | KO | 6 | 6 | 3 |
|  | Su_EPFL9*-*2a | OE | 8 | 8 | 5 |
|  | Su_EPFL9*-*2b | OE | 8 | 8 | 4 |
| **‘Syrah’** | WT |  | 5 | 5 | 3 |
|  | Sy_EPFL9*-*2a | KO | 8 | 8 | 5 |
| **‘Kober 5BB’** | WT |  | 10 | 10 | 10 |
|  | Ko_*epfl9-*2a | KO | 8 | 8 | 5 |
|  | Ko_*epfl9-*2b | KO | 8 | 8 | 5 |

**Table S5** Plants used in water stress experiment 2. For each irrigation regime (well-water, moderate water stress and severe water stress) the number of biological replicates involved is indicated.
